# Exploring molecular interactions between yeast topoisomerase I and RNA polymerase III subunit, Rpc82 using an *in-silico* approach

**DOI:** 10.3389/fbinf.2026.1897635

**Published:** 2026-10-09

**Authors:** Prakash Nandi, Izaz Monir Kamal, Saikat Chakrabarti, Sugopa Sengupta

**Affiliations:** 1 Department of Life Sciences, Presidency University Kolkata, Kolkata, India; 2 Structural Biology and Bioinformatics Division, CSIR Indian Institute of Chemical Biology, Kolkata, India; 3 Academy of Scientific and Innovative Research (AcSIR), Ghaziabad, India

**Keywords:** protein-protein interaction, RNA polymerase III, Rpc82, topoisomerase I, transcription-topology connection

## Abstract

**Introduction:**

The process of DNA transcription leads to the generation of torsional stress, which must be resolved for smooth progression of the transcription machinery. In Saccharomyces cerevisiae, DNA topoisomerase I (Top I), a type IB topoisomerase, plays a critical role in relaxing supercoils and mitigating the topological strain associated with transcription. While several proteins from the transcription machinery have been reported to interact with yeast Top I, the precise structural domains involved and the detailed features of the interaction interfaces remain largely unexplored. This gap is partly due to the absence of a complete three-dimensional structure of the full-length enzyme, which hinders structure-based computational analyses of its interactome.

**Methods:**

A full-length model of yeast Top I was generated using a domain-based modeling approach and evaluated using structural validation metrics and comparative analyses with homologous structures. Leveraging this model, we investigated a potential interaction between Top I and Rpc82, a key subunit of RNA polymerase III involved in transcription of small non-coding RNAs such as tRNAs and 5S rRNA. The interaction was evaluated using an integrative computational approach combining molecular docking, binding free-energy calculations, and molecular dynamics simulations.

**Results:**

The analyses identified a putative interaction interface involving the WH2–WH3 region of Rpc82 and primarily the D1 and D3 domains of Top I. Molecular dynamics simulations indicated that the predicted Top I–Rpc82 complex remained structurally stable under the simulated conditions. The calculated binding energy-to-surface-area ratio of the complex was comparable to benchmark values for biologically relevant heterodimeric protein interfaces, supporting the plausibility of the predicted interaction.

**Discussion/Conclusions:**

Overall these findings provide a structural framework for future experimental validation of Top I interactions, paving the way for mechanistic insights into its regulatory functions and contribution to genome maintenance and stability.

## Graphical Abstract

**Figure:**
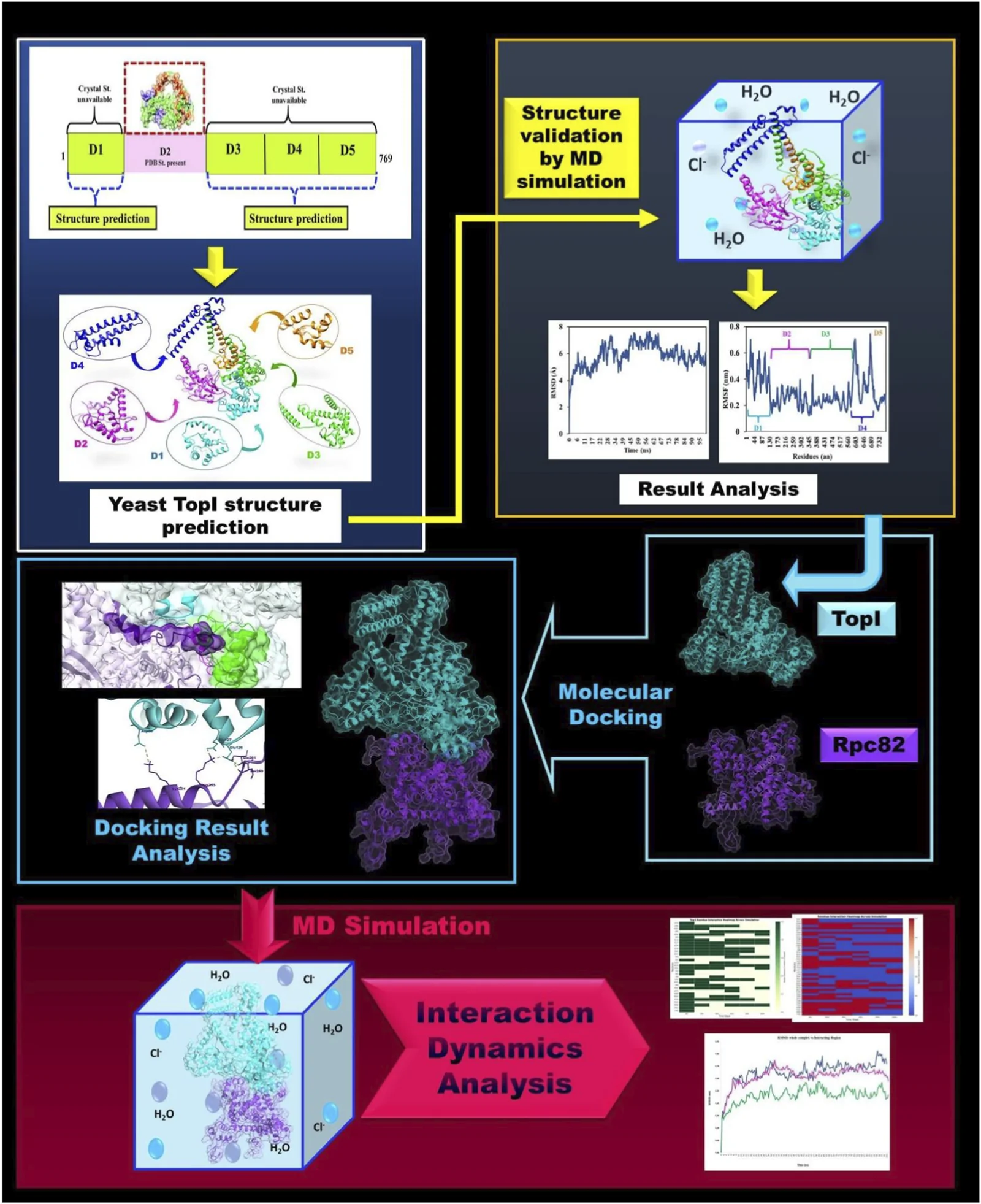

## 1 Introduction

During transcription, RNA polymerase generates positive supercoils ahead and negative supercoils behind the transcription bubble, which must be resolved to allow efficient elongation (Liu and Wang, 1987; Wu et al., 1988). RNA polymerase often gets hindered owing to accumulation of excess DNA supercoils and formation of R-loops (DNA-RNA hybrids) (Chedin and Benham, 2020; García-Muse and Aguilera, 2019; O’Connell et al., 2015). The level of supercoiling has a direct impact on promoter accessibility to transcription factors and RNA polymerase binding (Chatterjee et al., 2021). Topoisomerase I (Top I), a type IB topoisomerase, transiently cleaves a single-strand of DNA to relax both positive and negative supercoils, thereby alleviating transcription-associated topological stress (Brill and Sternglanz, 1988; Kim and Wang, 1989; Megonigal et al., 1997; Pommier et al., 2016; Takahashi et al., 2011; Tuduri et al., 2009; Wang, 1996). Direct physical interactions between RNA polymerases and DNA topoisomerase I have been reported to contribute to transcriptional regulation across diverse systems, ranging from bacteria to humans. The β′ subunit of bacterial RNA polymerase was shown to physically interact with the C-terminal domain of bacterial topoisomerase I, positioning the enzyme to relieve transcription-induced negative supercoiling and thereby facilitating efficient transcription elongation (Cheng et al., 2003). Unlike bacteria harbouring a single RNA polymerase, eukaryotic RNA polymerases exhibit division of labour wherein RNA polymerase I transcribes rRNA, RNA polymerase II transcribes mRNA and RNA polymerase III transcribes tRNA, 5srRNA, and snRNA (Woychik et al., 1990). Among these, physical interaction between RNA polymerase II and Top I have been reported from *Drosophila* and human systems (D’Arpa et al., 1988; Wu et al., 2010). During active transcription, RNA polymerase II-Top I interaction helps to recruit Top I and stimulate the latter’s relaxation activity, which in turn facilitates transcription process by managing DNA topology (Baranello et al., 2016). Although RNA polymerase II-Top I connection is well established, the physical link between Top I and other two eukaryotic RNA polymerases remain underexplored. Few studies have indicated a genetic and functional interaction between Rpa34 subunit of RNA polymerase I and yeast Top I (Collins et al., 2007; Costanzo et al., 2016; Wilmes et al., 2008). On the other hand, limited information exists on RNA polymerase III-Top I interaction till date. Human Top I has been reported to promote RNA polymerase III-mediated transcription reinitiation and termination processes (Wang and Roeder, 1998a). In budding yeast, two independent high throughput studies based on affinity chromatography followed by mass-spectrometric analyses reported physical interaction between Top I and Rpc82 subunit of RNA polymerase III (Chymkowitch et al., 2017; Ho et al., 2002). However, detailed characterization of the yTop I-Rpc82 interaction has remained elusive till date.

Structurally, Yeast RNA polymerase III (Pol III) comprises seventeen subunits, including five shared across all three RNA polymerases (Cuevas-Bermúdez et al., 2017; Carles et al., 1991; McKune et al., 1995; Rubbi et al., 1999; Shpakovski et al., 1995; Woychik et al., 1990). Among its unique components, Rpc82 forms a heterotrimer with Rpc34 and Rpc31 subunits to participate in the pre-initiation complex. This Rpc82–34–31 trimeric module aids in DNA unwinding to form the open complex (Bartholomew et al., 1993; Brun et al., 1997; McKune et al., 1995; Thuillier et al., 1995; Werner et al., 1992). Structurally, Rpc82 consists of four winged-helix domains (WH1–WH4 corresponds to the residue 31-123, 124-283, 284-476, 477-566 respectively) and a C-terminal coiled-coil (CC) domain (residue 567–654). It anchors to the clamp domain of the Pol III cleft and interacts with duplex DNA downstream of the transcription bubble, contributing to transcription complex stability (Khoo et al., 2018). A disordered insertion within its WH3 domain facilitates interaction with Brf1, a TFIIB-related transcription factor, during pre-initiation complex formation (Khoo et al., 2018), positioning Rpc82 as a key mediator of protein–protein contacts within the Pol III pre-initiation complex. It also helps in stabilizing the transcription bubble in association with Rpc34 (Carter and Drouin, 2010). Given its role in transcription initiation and its positioning near the DNA entry channel, Rpc82 is well placed to interact with other regulatory factors such as Top I. Pol III drives very high levels of transcription of tRNA and other genes, which are short and support rapid polymerase recycling. Repeated transcription cycles can generate torsional strain if not efficiently relaxed (Dieci et al., 2013). In budding yeast, loss of Top I was shown to enhance R-loop accumulation at highly transcribed Pol III loci, including tRNA genes, owing to unresolved torsional stress which in turn impaired transcription elongation and pre-tRNA synthesis (El Hage et al., 2014). These findings suggest that a direct physical and functional partnership between Pol III and Top I may be important for coordinating transcription to ensure efficient tRNA biogenesis.

Understanding protein–protein interactions (PPIs) is essential for deciphering cellular functions and regulatory mechanisms. Recent advances in bioinformatics have significantly enhanced the accuracy and speed of structural and interactome predictions, enabling rapid *in silico* analysis (Macalino et al., 2018). In this study, we investigated the molecular interaction between yeast DNA topoisomerase I (yTop I) and RNA polymerase III (Pol III) using computational approaches. Due to the lack of a full-length 3D structure of yTop I, we first constructed structural models of the individual domains of yTop I, using template-based modeling and validated them using multiple structural parameters. The selected individual-domain models were subsequently assembled into a full-length yTop I structure. To further assess their structural plausibility, the models for individual-domains and full-length yTop I were compared with reference distributions derived from high-resolution X-ray crystallographic structures of eukaryotic proteins. The selected yTop I model was subsequently evaluated for structural stability through molecular dynamics (MD) simulations. This model was then used to explore its interaction with Rpc82, a key subunit of Pol III. Using combinatorial protein–protein docking, we modeled the yTop I–Rpc82 complex and selected the most favourable binding pose. MD simulations and binding free energy calculations suggest a stable interaction between the two proteins. Comprehensive bioinformatic analyses provide preliminary insights into how yTop I could engage in interaction with the Pol III subunit Rpc82. Together, these observations offer a tentative structural framework for the Top I–Pol III interaction in budding yeast, which can guide future experimental validation studies. Given the evolutionary conservation of both proteins, a similar interaction may exist in humans, although this remains to be established. The Top I–Pol III interface in human counterpart could represent a potential site for regulating Pol III activity, particularly in contexts of diseases such as cancer where Pol III transcription is often dysregulated (Sun et al., 2025). However, significance and potential of this interaction as a druggable target will require further experimental validation.

## 2 Materials and methods

### 2.1 Identification of potential interactor of yTop I from yeast RNA polymerase III complex

To explore the connection between Topoisomerase I and transcription, initial information on physically interacting protein partners of yeast Topoisomerase I (yTop I) was compiled from multiple protein–protein interaction databases and literature sources, including the *Saccharomyces* Genome Database (Cherry et al., 1998), BioGRID (Stark et al., 2006), STRING (von Mering et al., 2003) and PubMed (https://pubmed.ncbi.nim.nih.gov). To identify transcription-related interactors, candidates were filtered based on nuclear localization, direct involvement in the transcriptional machinery, with the additional requirement of available 3D structural information to enable *in silico* analyses of the potential protein partner(s). Based on these criteria, the Rpc82 subunit of RNA polymerase III was finally selected, supported by evidence from two independent high-throughput studies in budding yeast, employing affinity chromatography coupled to mass spectrometry (Chymkowitch et al., 2017; Ho et al., 2002). Both these studies consistently identified Rpc82 as a physical interactor of Top I, suggesting a potential functional association between Top I and Pol III transcription machinery in budding yeast.

### 2.2 Template-based modeling of full-length yeast Top I

The full-length amino acid sequence of yTop I (UniProt ID: P04786) was retrieved from the *Saccharomyces* Genome Database and analyzed using the InterPro server (Mulder and Apweiler, 2008) to identify domain-specific secondary structural elements. Based on this analysis, yTop I was divided into five segments namely the N-terminal domain (D1, residues 1–142), DNA-binding domain (D2, residues 143–363), core domain (D3, residues 364–589), linker region (D4, residues 590–698), and C-terminal catalytic domain (D5, residues 699–769). Each segment was then subjected to molecular modelling individually, using multiple protein model prediction programs such as RoseTTAFold (Baek et al., 2021), I-TASSER (Bowie et al., 1991; Yang and Zhang, 2015), TrRosetta (Du et al., 2021), Ab-initio (Hardin et al., 2002; Pearce et al., 2022), and DeepFold (Lee et al., 2023). The crystal structure of the D2 domain is available (PDB ID: 1OIS) and was therefore excluded from the modelling exercise (Lue et al., 1995). However, the available D2 domain structure lacks the coordinates of two residues, Ser253 and Gln254, which were subsequently incorporated during the construction of the full-length yTopI model. For each of the remaining segments (D1, D3, D4, and D5), the top five models generated by each tool were selected based on the respective server-specific scoring criteria. These models were then considered for further quality assessment using the SAVES server (https://saves.mbi.ucla.edu), considering ERRAT scores (Colovos and Yeates, 1993), Verify3D parameters (Eisenberg et al., 1997), Ramachandran plot of stereochemical acceptance and high DOPE score with GA341 score of 1 (Supplementary Table S1). As the predictive performance of each method may vary across distinct structural regions of yTop I, the optimal model for each yTop I domain was then selected from the respective modeling approaches based on a comparative assessment of multiple structural scoring parameters (Supplementary Table S2). The crystallographically resolved D2 domain (PDB ID: 1OIS) and the selected models of the remaining four regions were then assembled into a full-length structure using the advanced template-based modeling method of MODELLER (Eswar et al., 2006). The coordinates of the two missing D2 domain residues, Ser253 and Gln254, in PDB ID 1OIS were incorporated into the full-length model using the LoopModel program in MODELLER. The RMSD of this region was subsequently assessed against the AlphaFold-predicted model (AF-P04786-F1). The best model of assembled full-length yTop I was selected from all the predicted structures based on the validation metrics, including ERRAT score, Verify3D, and Ramachandran favoured stereochemical plots (Hollingsworth and Karplus, 2010; Ramachandran et al., 1963) using the SAVES server (https://saves.mbi.ucla.edu). The selected model was subsequently refined through energy minimization to improve its structural quality.

### 2.3 Quality assessment and validation of predicted individual domains and full-length yTop I model

To evaluate the quality of the selected yTop I domain models and full-length structure, their structural parameters were compared with those of proteins with comparable sequence lengths retrieved from the RCSB PDB database (https://www.rcsb.org/). Due to the variation in polypeptide lengths among yTop I domains, high-resolution eukaryotic protein structures with comparable residue numbers (±10) were selected as references for the corresponding yTop I domains. These structures were analysed using ERRAT, Verify3D, and Ramachandran plot parameters. The obtained values were compared with a reference dataset comprising eukaryotic protein structures (42 for D1, 21 for D3, 51 for D4, 23 for D5, and 20 for full-length yTopI) determined by X-ray crystallography at a resolution of ≤3 Å. These benchmark values were subsequently used to evaluate the quality of the individual domain models and the full-length yTop I structure.

Structural studies have shown that despite sequence divergence, Type IB topoisomerases share a conserved core fold and catalytic mechanism (Baker et al., 2009; Cheng et al., 1998). To benchmark our model, it was compared with the human Topoisomerase I (hTop I, PDB ID: 1K4T), a structurally and functionally conserved orthologue. Sequence identity was checked between yeast and human Topoisomerase I, followed by the superimposition of the predicted yTop I model on hTop I model to assess the structural comparability of yTop I segments/domains that share higher sequence identity with their human counterparts.

To further ensure the reliability and robustness of the predicted full-length yTop I structure, a comparative analysis was performed using models generated from multiple structure prediction platforms. GalaxyWEB (Ko et al., 2012) and Protenix (Chen et al., 2025) servers were used to predict the yTop I full-length model by TBM (Template Based Modeling) approach. The top five models from each server, along with the AlphaFold-predicted yTop I structure, were systematically analysed and compared with the selected full-length model. This cross-platform comparison enabled benchmarking of model quality. QMEAN (Qualitative Model Energy ANalysis) is a composite scoring function used to estimate the global and local quality of protein structural models (Benkert et al., 2011). QMEAN scores were calculated for all the models and compared with the score obtained for our predicted model for yTop I.

### 2.4 Interaction profiling of yTop I and Rpc82 subunit of RNA polymerase III

To explore the interaction between yTop I and Rpc82, the predicted full-length yTop I model and the available yeast Rpc82 structure (PDB ID: 6EU0) (Abascal-Palacios et al., 2018) were used. Two molecular docking strategies, blind and targeted docking, were employed to characterize the interaction between yTop I and Rpc82. Blind docking was first performed using the full-length yTop I model and Rpc82 followed by targeted docking with the Rpc34/Rpc160-binding residues on Rpc82 masked to prevent interference. In parallel, domain-wise targeted docking was performed for individual yTop I domains (D1–D5) against full-length Rpc82, after masking the Rpc34/Rpc160-interacting regions of Rpc82 (Supplementary Table S3). Comparative analysis of all the docking solutions was performed to identify the most plausible yTop I–Rpc82 interaction interface.

Briefly, the blind docking was performed using the ClusPro (Kozakov et al., 2017), HDOCK (Yan et al., 2017), and, HADDOCK (Honorato et al., 2024) servers to identify the possible interaction surface(s) for both the proteins. The top ten docking models from each server were selected based on server-specific criteria. Binding energies and interaction interfaces were analysed using PDBePISA (Krissinel and Henrick, 2007) and visualized by Biovia Discovery Studio (2021). For each docking model, the percentage contribution of each domain of Rpc82 to the total number of intermolecular non-covalent interactions with yTop I was calculated and compared across poses to pinpoint the most probable interaction surface on Rpc82 protein. A similar analysis was conducted to identify the Rpc82 binding interface on yTop I as well. The targeted docking of full length yTop I and its individual domains at accessible regions of Rpc82 was performed using Cluspro (Kozakov et al., 2017) and HDOCK (Yan et al., 2020) servers, followed by binding energy estimation via PRODIGY (Xue et al., 2016). Interaction profiling and visualization were again carried out using PDBePISA and Biovia Discovery Studio. Two complementary computational approaches (PDBePISA and PRODIGY) were employed as they provide different and non-interchangeable information on the protein–protein interface. PRODIGY provides a contact-based prediction of protein–protein binding affinity, counting intermolecular contacts reported as an estimated binding free energy, whereas PDBePISA characterizes macromolecular interfaces and reports interface-associated parameters such as solvation-energy gain, buried surface area, hydrogen bonds, salt bridges, and assembly stability.

### 2.5 Comparative analysis of yTop I-Rpc82 binding interactions with known protein complexes

To further assess the quality of the selected yTop I–Rpc82 docking model, the ratio of binding energy to interaction surface area was calculated. The obtained value was then compared with those of established eukaryotic heterodimeric protein–protein complexes resolved by X-ray crystallography (≤3 Å resolution) and having comparable binding energies and interaction surface areas, following a previously reported method (Kastritis et al., 2011). Interaction surfaces were calculated using the EMBL PDBsum program (Laskowski et al., 2018), while binding energy (ΔG) values were estimated using the PRODIGY server (Xue et al., 2016). This comparative analysis was performed to validate the docking model by benchmarking its interaction energy characteristics against known protein-protein complex standards.

### 2.6 MD simulations

MD simulations offer valuable insights into protein stability, flexibility, and conformational dynamics. The predicted yTop I model was subjected to MD simulation analysis using GROMACS version 2024 (Van Der Spoel et al., 2005; Vieira et al., 2023). AMBER99SB protein force field (Lindorff-Larsen et al., 2010) was utilized to define the topology file. The protein-in-water complex was accommodated in a box of 2081.95 nm^3^ cubes. Charge neutralization was achieved by the application of 12 Cl^-^ ions. Steepest descent (Petrova & Solov’ev, 1997) and conjugate gradient algorithms (O’Leary, 1980) were used for energy minimization, followed by the system equilibration in the NVT and NPT ensembles at 300 K for a 0.1 ps time constant. Final MD run was performed with a step size of 2 fs. Stability and dynamics of the protein were assessed by calculating different parameters like Root Mean Square Deviation (RMSD), Root Mean Square Fluctuation (RMSF), Solvent Accessible Surface Area (SASA), Radius of Gyration (Rg) *etc.*, for the simulation trajectory.

Similarly, to assess the structural dynamics and stability of the selected Top I-Rpc82 docking complex, MD simulation studies were conducted for 200 ns using the GROMACS platform (Lemkul, 2019; Van Der Spoel et al., 2005). AMBER99SB protein force field (Lindorff-Larsen et al., 2010) was applied to perform these studies. The protein -in-water complex was measured to be occupying a cubic volume of 5505.45 nm^3^, which was made neutral by applying 24 Cl^-^ ions. Energy minimization of the system was performed by steepest descent (Petrova & Solov’ev, 1997) and conjugant gradient algorithms (O’Leary, 1980), followed by the system equilibration in the NVT and NPT ensembles at 300 K at a 0.1 ps time constant. The MD run followed the 2 fs step size to attain the total of 200 ns simulation. These MD simulations were performed in triplicate to ensure statistical robustness of the observed trends and to detect any significant discrepancies between simulation runs.

The PDBePISA server (Krissinel and Henrick, 2007) and Biovia Discovery Studio software (https://discover.3ds.com/discovery-studio-visualizer-download) were employed to identify the interacting residues of the two proteins during the simulation course by choosing different coordinate frames at regular intervals from one representative simulation trajectory. The major interacting zone during the simulation was also determined, and its interaction dynamics were assessed by calculating the RMSD, RMSF, Rg, and SASA values of the interacting residues/surface, followed by extrapolation of the values on the respective graphs of the whole complex. Further analysis of the yTop I–Rpc82 interaction interface during the simulation was performed by determining the buried and interaction surface areas, as well as the number and types of intermolecular interactions, using the COCOMAPS 2.0 server (Chawla et al., 2025). Structural analysis of the simulation trajectory at 10-ns intervals using COCOMAPS 2.0, together with identification of interacting residues using HADDOCK and PDBePISA, provided information regarding the interaction interface formed between yTop I and Rpc82.

### 2.7 MM-PBSA analysis of yTop I-Rpc82 complex stability

Protein-protein interaction energetics were quantified using the Molecular Mechanics Poisson-Boltzmann and Surface Area (MM-PBSA) methodology (Genheden and Ryde, 2015). The calculation was performed using the gmx_MMPBSA interface module, which extracts snapshots from the MD trajectory and estimates the free energy of the complex, as well as those of the individual interacting components, on a frame-by-frame basis.

The energy of the protein–protein complex (G_complex_) was calculated for each frame of the MD trajectory using the trajectory extraction module (fit.xtc), according to the following equation:

$$G_{\text{complex}}=E_{\text{MM}+}G_{\text{polar}}+G_{\text{nonpolar}}$$

where E _MM_ represents the molecular mechanics energy, comprising of bonded (E _bonded_) and non bonded (E _nonbonded_) interactions, while G _polar_ and G _nonpolar_ denote polar and nonpolar energy contributions, respectively. Summated result of the mentioned parameters of all the frames were finally extracted to calculate the binding free energy of the complex (ΔG _Bind_). All these values were subsequently summed to obtain the total free energy of the complex.

The binding free energy was calculated using the fundamental thermodynamic equation

$$\Delta G=\Delta G_{\text{complex}}–\;\left(\Delta \text{PE}+\Delta \text{PE}\prime \right)$$

where ΔG _complex_ represents the complex free energy, and ΔPE and ΔPE' denote individual protein free energies. The comprehensive MM-PBSA binding free energy equation,

$$\Delta G_{\text{Bind}}=\Delta E_{\text{MM}}+\Delta G_{\text{solv}}-T\Delta S$$

systematically decomposes molecular interaction energetics into molecular mechanics energy (ΔE _MM_), solvation free energy (ΔG _Solv_), and entropic contributions. Molecular mechanics energy was further dissected into bonded (ΔE _bonded_) and non-bonded (ΔE _nonbonded_) components, which were further resolved into van der Waals (ΔE _vdW_) and electrostatic (ΔE _elec_) interactions. Solvation free energy (ΔG _Solv_) was analysed by adding polar (ΔG_polar_) and non-polar (ΔG _nonpolar_) contributions. MD simulations were performed using GROMACS with a 200 ns trajectory, generating 400 snapshots at 500 ps intervals under standardized conditions with 0.154 M salt concentration, dielectric constants of 1.0 (internal) and 80.0 (external). The gmx MM-PBSA (Genheden and Ryde, 2015) computational interface enabled binding free energy estimations by incorporating an interaction entropy (I.E.,) module (Duan et al., 2016) to directly quantify entropic contributions from MD simulation ensembles.

The final binding free energy was determined using the following equation

$$\Delta G_{\text{binding}}=\Delta H\;–\;T\Delta S$$

where ΔG binding represents Gibbs free energy of protein-protein interactions, ΔH accounts for molecular interaction enthalpy, and TΔS captures conformational entropy. This methodology provides a rigorous computational framework for investigating protein-protein interaction energetics at atomic resolution, combining trajectory generation, snapshot analysis, energy component decomposition, and entropic contribution assessment.

## 3 Results

### 3.1 Structural modeling of full-length topoisomerase I from budding yeast

Three-dimensional (3D) structural information is essential for *in silico* molecular docking studies. While the structure of Rpc82 is available in the RCSB-PDB (PDB ID: 6EU0), yeast Topoisomerase I (yTop I), a 769-residue protein, lacks a complete structure. The only crystal structure available (PDB ID: 1OIS) covers the DNA-binding domain (residues 141–363), leaving the rest of the protein unresolved. Even the AlphaFold prediction (AF-P04786-F1) shows very low pLDDT score (<50) for the N-terminal domain (NTD) of y Top I, indicating low confidence in the predicted structure of this region. Top I-NTD is known to mediate interactions with several cellular proteins such as Rpb1 (Baranello et al., 2016), and Sir2 (D’Alfonso et al., 2016). Thus, it was necessary to build a model for full-length yTop I to enable investigation of its interaction with Rpc82. Initial homology modeling using SwissModel program (Waterhouse et al., 2018) based on 51.69% sequence identity with the human orthologue, failed to capture the first 128 N-terminal residues and a significant C-terminal stretch. Therefore, to enable reliable interaction studies, an alternative modeling strategy was adopted to generate structural information for key regions of yTop I, including the NTD (D1) and linker region (D4), which were missing from existing AlphaFold and homology-based models.

To ensure unbiased 3D structure prediction, each yTop I domain (D1, D3, D4, D5) was modeled using multiple programs and validated using previously described rigorous criteria. All selected models are based on structural accuracy, with ERRAT scores above 95% and high Verify3D scores (exceeding 85% in most cases), indicating strong sequence–structure compatibility. Ramachandran plot analysis revealed over 91% residues in favored regions with minimal presence in disallowed zones (Supplementary Figure S1; Supplementary Table S2). The validated domain models were then assembled using MODELLER to generate the full-length yTop I structure (Figure 1; Supplementary Table S1). The final individual domain models and full-length model were selected based on optimal structural validation metrics. Although the individual domain models exhibited ERRAT scores above 95%, the assembled full-length model showed a slightly lower ERRAT score of 93.43%, together with Verify3D score of 86.35%, 94.3% of residues in the Ramachandran favored region and only 0.1% in the disallowed region, supporting the overall structural reliability of the model (Supplementary Table S1; Supplementary Table S2). The modest reduction in ERRAT score following domain assembly may reflect minor local structural strain or suboptimal non-bonded contacts introduced at the newly constructed inter-domain junctions and linker regions. The D2 region of the full-length model includes Ser253 and Gln254 residues and exhibits a low RMSD of 0.343 Å relative to the corresponding AlphaFold model (AF-P04786-F1) for the corresponding region (Lys245–Arg260).

**FIGURE 1:**
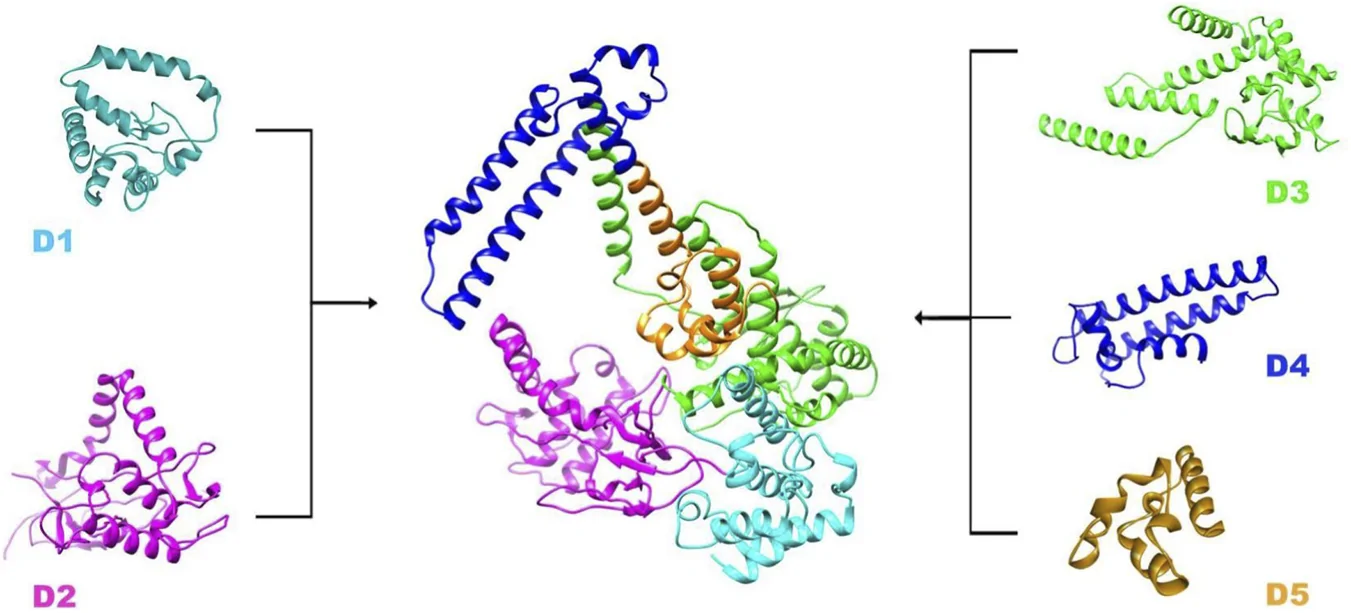
Domain-wise representation of the modeled Topoisomerase I from budding yeast. The full-length budding yeast Topoisomerase I (yTop I) structure was assembled by integrating the predicted models of domains D1, D3, D4, and D5 with the experimentally resolved D2 domain (PDB ID: 1OIS). The central image depicts the assembled full-length yTop I model, with individual domains distinctly color-coded as D1 (cyan), D2 (purple), D3 (green), D4 (blue), and D5 (orange), illustrating their spatial organization within the modeled structure.

### 3.2 Comparative structural validation and benchmarking of yTop I domains and full-length model

The quality of the predicted regions of yTop I was evaluated through comparative analysis against structural parameters derived from high-resolution protein crystal structures. The D2 segment was excluded from this analysis due to the availability of its experimentally resolved crystal structure (PDB ID: 1OIS). For each of the remaining segments (D1, D3, D4, and D5), at least 20 eukaryotic proteins with similar residue lengths were selected from the RCSB-PDB database. These high-resolution crystallographic protein structures were then assessed using ERRAT, Verify3D, and Ramachandran plot parameters, and average values were calculated to establish benchmark ranges for comparison with the predicted model for the corresponding yTop I domain(s). A total of 42 structures were used to benchmark the D1 domain of yTop I (Supplementary Table S4), 21 structures for D3 (Supplementary Table S5), 51 structures for D4 (Supplementary Table S6), and 23 protein structures for D5 domain (Supplementary Table S7). All three evaluated parameters for both the individual domains and the full-length yTop I model were within the benchmark ranges and, in most cases, exceeded the corresponding average reference values (Figures 2A–E) (Supplementary Table S8).

**FIGURE 2:**
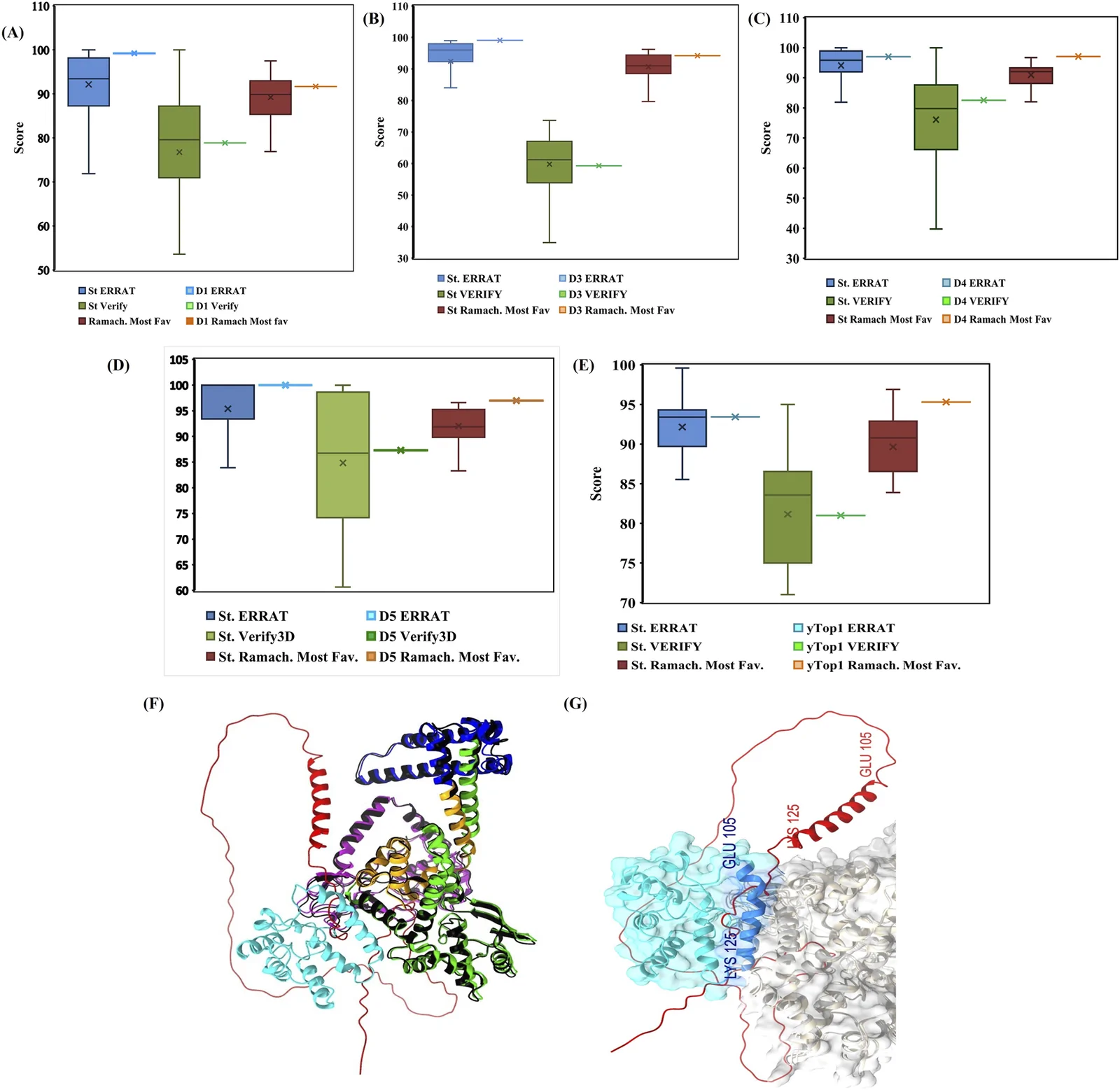
Comparison of structural parameters of the predicted individual domains and full-length yTop I model with standard (St) values derived from high-resolution PDB structures and the AlphaFold model of yTop I. **(A–E)** ERRAT, Verify3D, and Ramachandran most favoured residue percentage values were compared, darker shades indicating the average score of multiple protein structures to develop standard and lighter shades indicating the same parameter scores of selected individual domains **(A–D)** or full-length model of yTop I **(E)**. St. Represents standard values for reference proteins. Crosses in the plots indicate mean values. Comparative whisker plots for **(A)** D1, **(B)** D3, **(C)** D4, **(D)** D5, and **(E)** the full-length yTop I model. **(F)** Superimposition of the predicted yTop I model (D1–cyan, D2–purple, D3–green, D4–blue, D5–orange) onto the AlphaFold-predicted structure (D1–red and dark grey for rest of the length) **(G)** Enlarged view of the D1 region from the predicted model (cyan), highlighting the resolved α-helix spanning residues 105–125 (dark blue), which corresponds to the only recognizable secondary structural element present in the D1 region of the AlphaFold model (shown in red).

To further validate the full-length model, a comparative analysis was performed against yTop I structures predicted by other servers, including GalaxyWEB, Protenix, and AlphaFold (Version 6). QMEAN score evaluates how “native-like” a predicted structure is by comparing statistical potentials derived from high-resolution experimental protein structures, wherein values closer to zero indicate better agreement with experimentally determined protein structures (Benkert et al., 2011). Based on QMEAN scores, along with ERRAT, Verify3D, and Ramachandran plot evaluations, the selected yTop I model exhibited overall quality comparable to, and in several assessment metrics better than, the models generated by the GalaxyWEB and Protenix servers (Supplementary Table S9). Although Protenix model five displayed a comparatively better QMEAN score, it was excluded from further consideration due to lower Verify3D scores, reduced residue percentage in the most favored regions of the Ramachandran plot, and the absence of a well-defined three-dimensional structure for the D1 domain in comparison to our full-length yTop I model. The AlphaFold model (Version 6) too lacked structural information for the D1 region and showed an overall QMEAN score of −1.08 and pLDDT score of D1 region below 50, placing it at the lower end of the quality range among the evaluated models. Our predicted model for full-length yTop I successfully met all the criteria in these comparative evaluations and was therefore chosen for downstream analyses.

The UniProt entry for yeast Topoisomerase I (yTop I, P04786) reveals conservation of key catalytic residues and domain architecture with its human counterpart. Sequence similarity analysis identified human Topoisomerase I (hTop I, PDB ID: 1K4T) as the closest homolog, with highest similarity in the D2, portion of D3 and D5 region of yTop I. Overall, yTop I shares 51.69% sequence identity and 45% similarity with hTop I. To ascertain whether our predicted model for yTop I model successfully captures the essential structural features of the enzyme, especially around the active-site tyrosine and DNA-binding motifs, a structural comparison was performed for our predicted model against the hTop I structure. Since the available structural model of the human Topoisomerase I (hTop I) protein lacks information on the N-terminal 173 residues, thus, D1 of yTop I could not be included in this comparison. Structural superimposition using RMSD values is a widely accepted method for assessing the quality of predicted protein models. RMSD measures the average distance between corresponding atoms of two superimposed structures following optimal alignment. In general, a lower RMSD value (<2 Å) indicates a closer structural match and therefore a more accurate model (Kufareva and Abagyan, 2011; Mechelke and Habeck, 2010). The predicted full-length yTop I model was superimposed onto the hTop I structure, yielding an overall RMSD value of 1.021 Å (Supplementary Figure S2A). Additionally, superimposition of yTop I domains D2–D5 onto the AlphaFold-predicted structure (Figure 2F) yielded an RMSD of 0.7 Å, indicating a very high degree of structural agreement between the two models. Notably, the AlphaFold model lacks well-defined secondary structural elements within the D1 region, which is characterized by pLDDT scores below 50, except for a single α-helix spanning residues 105–125. Importantly, our predicted full-length yTop I model successfully recapitulated this α-helical feature (Figure 2G), further supporting the structural reliability and accuracy of the model.

To further evaluate the individual domain models of yTop I, sequence and structural comparisons were performed against the human Topoisomerase I (hTop I). The D2 domain of yTop I shares 51.14% sequence identity and 46% similarity with residues 213–431 of hTop I, yielding the RMSD of 0.941 Å upon superimposition (Supplementary Figure S2B). Domains D3 and D5 showed 54.02% and 54.9% sequence identity, and 44% and 49% similarity with hTop I (PDB ID:1K4T) residues 432–663 and 695–765, respectively. Their RMSD values were 0.805 Å (Supplementary Figure S2C) and 1.29 Å (Supplementary Figure S2D). Overall, RMSD fluctuations for D2, D3, and D5 remained below 2 Å, confirming that the predicted *Saccharomyces cerevisiae* Top I model aligns very closely with the human Top I reference, particularly in the conserved regions. In contrast, the D4 linker region lacked sequence identity with any resolved eukaryotic topoisomerase structures in the RCSB-PDB, suggesting it may be unique to budding yeast or highly divergent. Given that linker regions in Topoisomerase I are generally flexible and often involved in transient interactions, structural comparison for D4 segment was performed using the AlphaFold-predicted model of yTop I as a reference. Superimposition yielded an RMSD value of 1.253 Å, (Figure 2F; Supplementary Figure S3).

### 3.3 Molecular dynamic simulations to assess structural dynamics of the yTop I model

MD simulations were performed to further assess the stability, flexibility and conformational dynamics of the predicted yTop I model. As shown in Figure 3A; Supplementary Figure S4A, the mean RMSD values across the triplicate MD simulations remained around 0.7 nm throughout the 200 ns simulation. The RMSD increased above 0.6 nm after approximately 50 ns but gradually declined toward 0.6 nm by 100 ns Following 100 ns, the RMSD exhibited a gradual increase accompanied by dynamic fluctuations. Although fluctuations in RMSD values were observed, these remained restricted to a relatively narrow range (∼0.6nm–0.7 nm) over the subsequent 100 ns, without any pronounced increase. This data suggests that the predicted yTop I structure maintained a relatively stable conformation throughout the 200ns simulation. RMSD plots of the individual runs are presented in Supplementary Figure S4B. RMSF analysis of individual amino acid residues (Figure 3B; Supplementary Figure S5) revealed comparatively higher fluctuations within the N-terminal domain (D1) and the linker region (D4), with values ranging between 0.6 and 1 nm. In contrast, the D2, D3, and D5 domains exhibited lower fluctuations (0.2–0.4 nm), indicating relatively reduced flexibility in these regions. Increased fluctuations, reaching nearly 1 nm, were observed in the N-terminal region (residues ∼50–90), while fluctuations of approximately 0.6 nm were observed in the linker regions. These higher RMSF values are consistent with the generally flexible nature of these regions in Top I. SASA values remained relatively consistent at ∼480 nm^2^ with a decreasing trend throughout the 200 ns simulation course (Figure 3C), suggesting stable surface exposure. The Rg values remained close to ∼3.55 nm throughout the simulation, with a slight decreasing trend after 160 ns (Figure 3D; Supplementary Figure S6), indicating overall structural compactness. Overall MD simulation results support the structural stability of the predicted yTop I model and suggest that it is suitable for subsequent protein–protein docking studies. The MD simulation was conducted primarily to validate the structural integrity and stability of the predicted yTop I structure.

**FIGURE 3:**
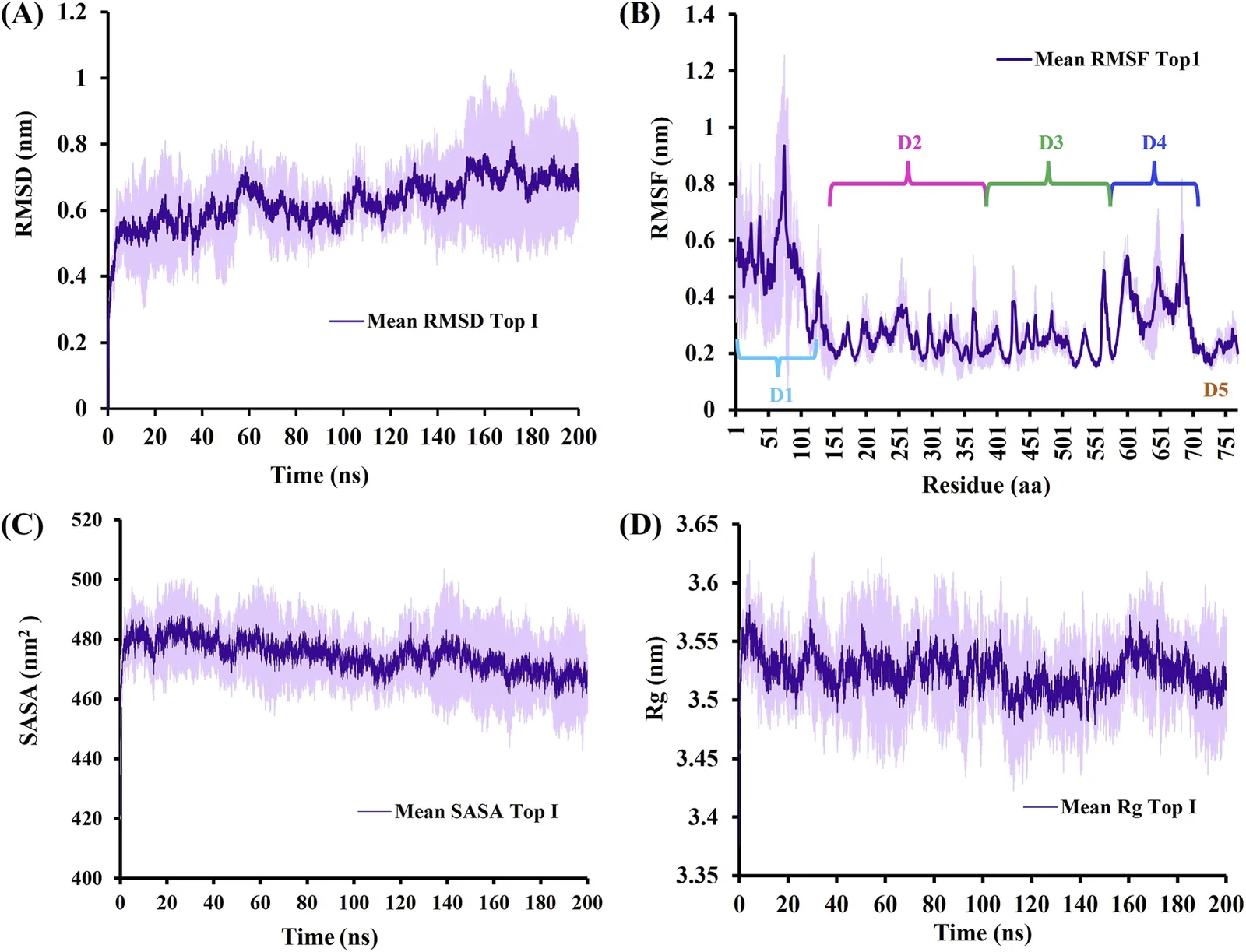
MD simulation analysis of predicted full-length Top I structure from budding yeast. **(A)** RMSD plot of the full-length Top I model during the simulation. **(B)** RMSF analysis showing residue-wise fluctuations across different regions of Top I. **(C)** SASA profile of the predicted model throughout the simulation. **(D)** Rg plot of Top I model. The dark violet line represents the mean values obtained from three independent replicate runs, while the shaded light violet area indicates the corresponding standard deviation of the MD parameters.

### 3.4 Interaction analysis of the yTop I–Rpc82 complex

In order to explore the molecular interactions between Rpc82 and yTop I, molecular docking was performed initially with full-length predicted yTop I and full-length Rpc82. Two distinct docking interfaces for yTop I were identified on the Rpc82 surface, one comprised of the WH2–WH3 regions and the second potential interface formed by the WH1–WH4/CC regions of Rpc82. Among the identified interfaces, the most favorable docking pose, characterized by a strong negative ΔG value, highlighted the WH2–WH3 region of Rpc82 as the primary interaction surface (Figures 4A,B; Supplementary Figure S7). This interface was prioritized for further analysis based on both energetic and structural considerations. Unlike the WH1–WH4 and CC domains, which are structurally committed to stable interactions within the Pol III core (notably with Rpc160 and Rpc34, as seen in the RNA polymerase III holoenzyme structure (PDB ID: 6EU0) (Abascal-Palacios et al., 2018; Hoffmann et al., 2015), the WH2–WH3 region remains spatially accessible (Supplementary Figure S8). Its peripheral location and involvement in transient interactions, such as its WH3 domain with Brf1 during pre-initiation complex formation make it a plausible docking site for regulatory proteins like Topoisomerase I. Moreover, selecting an interface outside the core-engaged domains avoids steric clashes and preserves the integrity of the Pol III assembly. Therefore, the best docking pose involving accessible regions i.e., the WH2–WH3 surface (Figures 4A,B; Supplementary Figure S7) was considered for further evaluation. Since the targeted docking strategy yielded results comparable to those obtained from the blind docking analysis and did not provide any additional insights, the corresponding data are not shown. Collectively, the WH2–WH3 region of Rpc82 represents a structurally viable and putatively relevant surface for Top I interaction, consistent with docking energetics and the known functional roles of Rpc82. As shown in Figures 4C–E, from the Top I side, *in silico* analysis indicates that discrete segments of both the D1 and D3 domains are involved in interactions with the WH2 and WH3 domains of Rpc82. In both full-length yTop I blind docking and domain-based targeted docking analyses, the region containing the missing D2 domain residues, Ser253 and Gln254, was found to be located away from the protein–protein binding interface.

**FIGURE 4:**
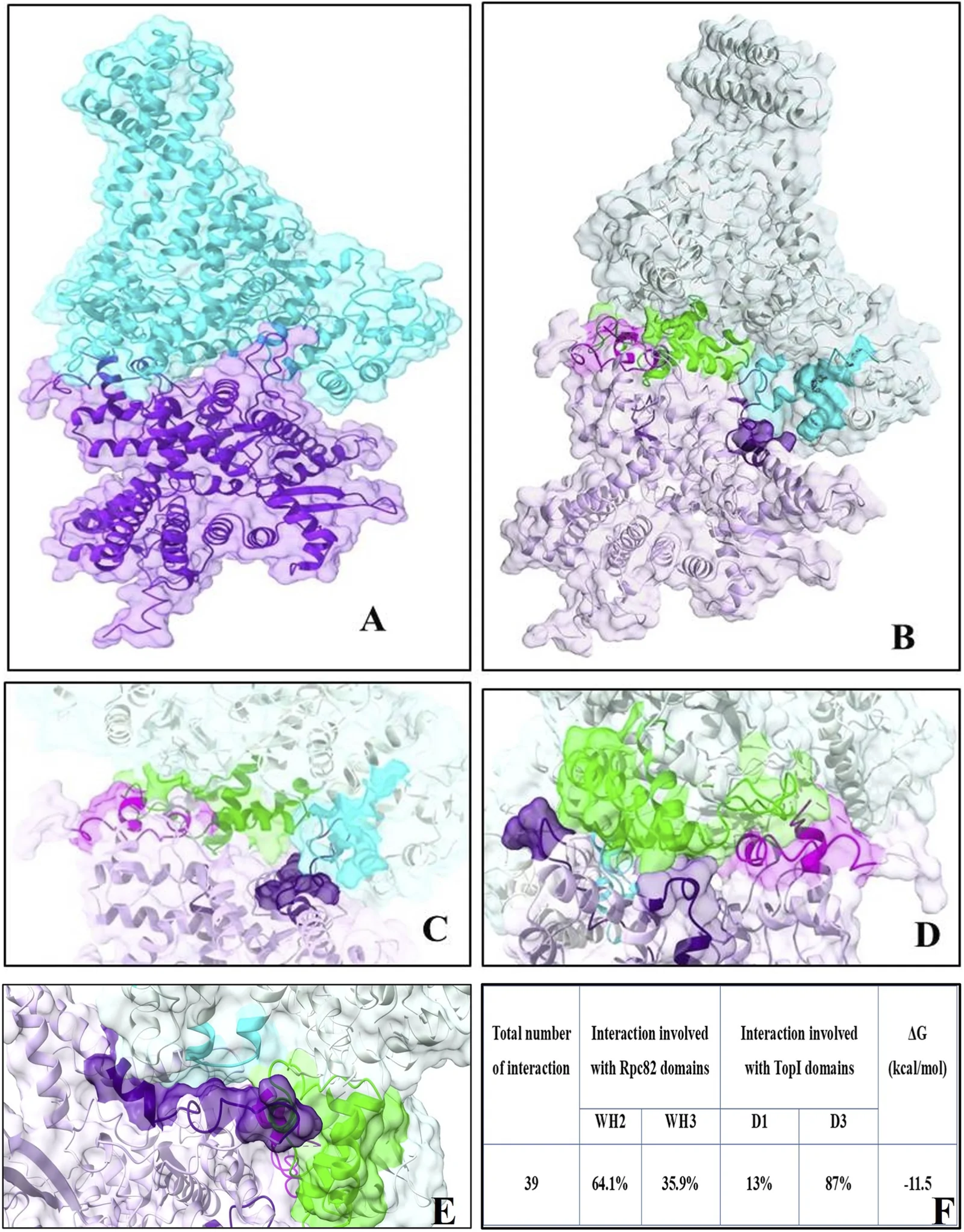
Structural analysis of the yTop I–Rpc82 docking complex and domain-specific interactions. **(A)** Selected docking model of yTop I (cyan) in complex with Rpc82 (violet). **(B)** Overall yTop I–Rpc82 docking complex, with the interacting surfaces highlighted in colour and the remaining portions of both proteins shown in grey. **(C–E)** Close-up views of the interaction interfaces involving yTop I domains D1 (cyan) and D3 (green) interacting with the WH2 (violet) and WH3 (purple) domains of Rpc82. The non-interacting surfaces of yTop I and Rpc82 are shown in faint blue and pale violet, respectively. **(F)** Schematic representation of the domain-wise involvement of Rpc82 in its interaction with full-length yTop I in the selected docking complex. Binding energy (ΔG in kcal/mol) was calculated using PDBePISA server.

To further support our findings and identify the most plausible interaction surface on Rpc82, a targeted docking approach was employed in parallel, using individual yTop I domains for docking against the accessible regions of Rpc82. Top-scoring docking models were selected based on binding free energy calculations from the PRODIGY server (Xue et al., 2016). The results summarized in Supplementary Table S3, indicate that domains D1, D2, D4, and D5 of yTop I predominantly interact with the WH2–WH3 region of Rpc82 (residues 140–196 and 272–369). Notably, the D3 domain of yTop I also appears to engage this surface, along with additional contacts at the WH1 domain. Among all domains, D1 and D3 showed comparatively stronger predicted binding affinities, suggesting a potentially more prominent role in the interaction within the computational framework. The convergence of both blind docking (full-length yTop I) and targeted docking using individual domains on the WH2–WH3 region of Rpc82 supports its putative relevance as a primary interaction interface. Furthermore, the WH3 domain’s reported interaction with Brf1 highlights its accessibility and functional flexibility, even within the Pol III holoenzyme (Khoo et al., 2018).

### 3.5 Binding free energy analysis of yTop I-Rpc82 complex

As outlined in the methods section, molecular docking of full-length yTop I with Rpc82 was performed using multiple servers. From each docking solution, the top ten scoring complexes were analysed to assess binding energies, interaction frequencies, and domain-specific contributions of Rpc82 towards complex stability. The selected docking model (Figures 4A; Supplementary Table S10- Cluspro 9) revealed that yTop I predominantly interacts with the WH2 and WH3 domains of Rpc82. This model exhibited a favorable binding energy of −11.5 kcal/mol, as determined by PDBePISA server (Figure 4F). The analysis further revealed the involvement of distinct Rpc82 surface regions and their domain-specific contributions to the interaction with yTop I. As shown in Figure 4F, a total of 39 intermolecular contacts were identified between yTop I and Rpc82. The relative contribution of the WH2 and WH3 domains of Rpc82 to the interaction interface was quantified by calculating the percentage of contacts formed by each domain relative to the total number of intermolecular contacts. Similarly, the contributions of the D1 and D3 domains of yTop I to the interaction interface were also estimated based on their respective percentages of intermolecular contacts with Rpc82. These analyses provided an *in silico* estimate of the relative contributions of the WH2 and WH3 domains of Rpc82 and the D1 and D3 domains of yTop I to the formation of the yTop I–Rpc82 complex.

The second predicted binding surface on Rpc82 (formed by WH1-WH4, CC domain) was not considered for further detailed analysis, as this region is involved in RNA polymerase III holoenzyme assembly, where interactions with core subunits would likely lead to steric hindrance. Interacting residue identification and heatmap analysis (Supplementary Figure S9; Supplementary Table S10) revealed domain-wise energy contributions and interaction percentages across Rpc82, further supporting that the WH2–WH3 region represents a more accessible and energetically favorable interface. This preference may be attributed to the pre-occupancy of the WH1–WH4–CC region by Rpc160 and Rpc34 during RNA polymerase III holoenzyme assembly, thereby limiting its accessibility for interaction with yTop I. From the perspective of yTop I, *in silico* mapping of interface residues suggests that amino acids from the D1 and D3 domains, along with a limited number of residues from the D2 domain (Supplementary Table S3), contribute to the interaction surface of Topoisomerase I.

### 3.6 Validation of yTop I-Rpc82 complex through comparative structural analysis

As described previously, the docking model in which yTop I preferentially interacts with the WH2–WH3 domains of Rpc82 was selected for further analysis due to its biological plausibility. To further assess the reliability of this predicted yTop I-Rpc82 complex, its protein–protein interaction energy and buried surface area were compared with a benchmark dataset of heterodimeric protein complexes from the RCSB-PDB (Supplementary Table S11). As shown in Figure 5A, the energy plot of the selected yTop I-Rpc82 docking complex demonstrated that the binding energy value calculated for the yTop I-Rpc82 complex (−12.7 kcal/mol, Supplementary Table S11), falls within the range observed for natural heterodimeric protein-protein interactions, indicating a stable and favorable interface. These results indicate that the selected yTop I-Rpc82 complex exhibits a stable and energetically favourable interaction. Similarly, the whisker plot of the energy/surface area value of the yTop I-Rpc82 complex closely aligns with benchmark values derived from well-characterized heterodimeric protein interactions (Figure 5B). This clustering within the biologically accepted range reinforces the notion that the predicted complex mimics natural protein–protein interfaces, further supporting the plausibility of the modeled interaction.

**FIGURE 5:**
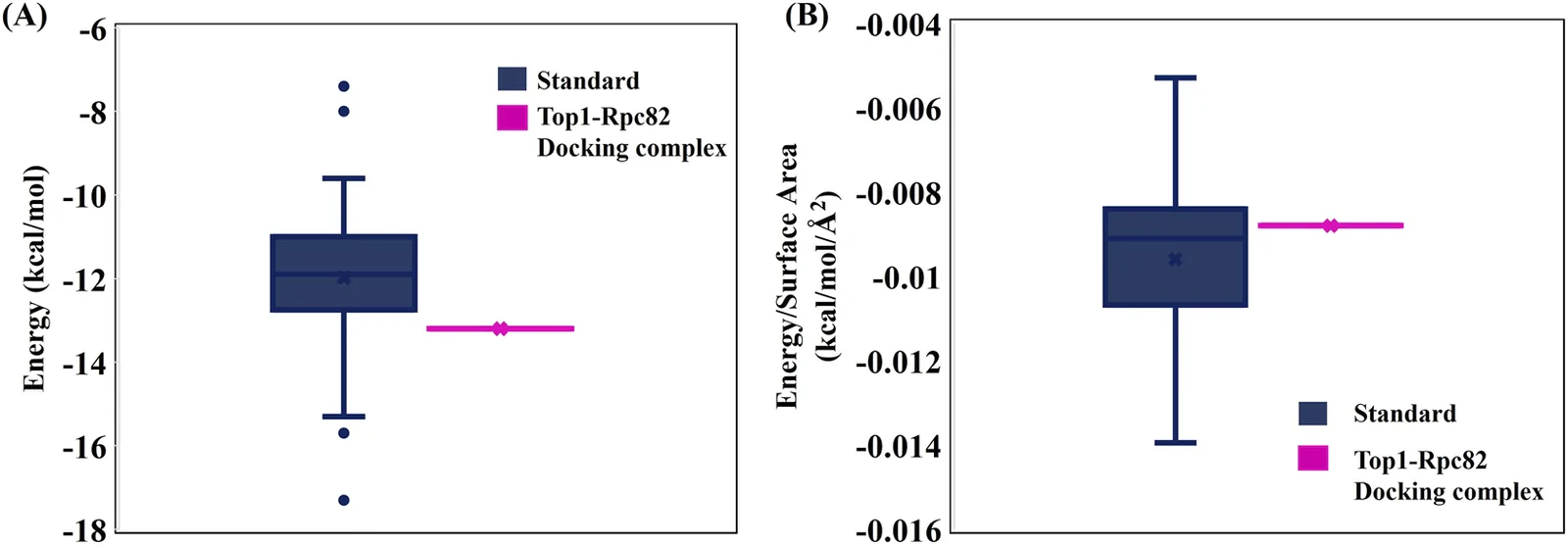
Comparative analysis of yTop I-Rpc82 docking complex against the reference dataset of experimentally validated heterodimeric protein complexes **(A)** Binding energy plot comparing the yTop I–Rpc82 docking complex with a reference dataset of experimentally validated heterodimeric protein complexes. The selected model shows energy values within the typical range observed for stable protein–protein interactions. The binding energy values for each complex were calculated using the PRODIGY server. **(B)** Whisker plot illustrating the energy-to-surface-area ratio of the yTop I–Rpc82 complex relative to benchmark heterodimeric interfaces.

### 3.7 MD simulation suggest stability of yTop I–Rpc82 complex

The overall interaction dynamics of the yTop I–Rpc82 complex were evaluated by analyzing multiple parameters across three independent MD simulation trajectories. RMSD analysis (Figure 6A; Supplementary Figure S10) showed initial deviations in the range of 0.6–0.7 nm, followed by a gradual increase to approximately 0.8 nm over the first 40–50 ns, indicative of an equilibration phase. Although minor fluctuations were observed, the RMSD values remained around 0.8 nm until the end of the initial 100 ns? During the subsequent 100 ns of the simulation, the RMSD values exhibited variations of approximately 0.2 nm and the mean RMSD value of the complex remained within the range of 0.9–1.0 nm between 100 ns and 200 ns Overall, the RMSD trajectory indicates that, despite conformational fluctuations, the yTop I–Rpc82 complex remained relatively stable throughout the simulation.The SASA profile of the protein–protein complex (Figure 6B) remained relatively constant throughout the simulations, with the solvent-accessible surface area ranging between 780 and 800 nm^2^ across the replicate runs. RMSF analysis was performed to assess residue-level flexibility within the yTop I–Rpc82 complex. In yTop I, the higher RMSF values were predominantly localized to regions known to exhibit intrinsic structural flexibility, whereas the rest of the protein displayed relatively lower RMSF values, indicating limited residue-level fluctuations (Figure 6C). In contrast, Rpc82 exhibited relatively higher and heterogeneous residue-level fluctuations across multiple domains, with RMSF values ranging from approximately 0.2–1.0 nm (Figure 6D). Moreover, as shown in Figures 6C,D, the interaction surfaces of yTopI formed by the D1 and D3 regions displayed distinct fluctuation profiles. The D3 interaction region exhibited relatively low fluctuations, with RMSF values of approximately 0.3 nm, whereas the D1 interaction region showed comparatively higher fluctuations, ranging from 0.4 to 1.0 nm. Thus, although both D1 and D3 contribute to the interaction interface with Rpc82, the D3-mediated interface appears more conformationally restricted, while the D1-mediated interface retains greater local flexibility. This difference is consistent with the overall RMSF profile of yTopI, where higher fluctuations were observed in portions of the D1 region and the more flexible D4 region. A similar pattern was observed for the Rpc82 interaction surface. The WH2 and WH3 regions, which form the interaction interface with yTopI, exhibited differential flexibility. While several residues within these regions showed relatively low fluctuations of approximately 0.4 nm, localized portions exhibited higher RMSF values of up to 0.8 nm. Thus, the Rpc82 interaction surface is not uniformly rigid, with certain segments of WH2 and WH3 displaying greater local flexibility than others. In comparison, higher fluctuations of nearly 1.0 nm were observed in the non-interacting WH4, cleft-loop (residues 550–563), and coiled-coil regions, indicating that the interaction regions of Rpc82 are comparatively more conformationally restrained. These observations highlight the interplay between stability at the binding interface and intrinsic flexibility in the other regions of the protein-protein complexes under dynamic physiological conditions. The buried surface area of the yTop I–Rpc82 complex remained relatively constant at approximately 30 nm^2^ during the initial phase of the simulation, followed by a gradual increase to ∼40 nm^2^ by the end of the 200 ns simulation. This increase suggests a greater extent of protein–protein interface burial during the latter phase of the simulation. Similarly, the Rg values obtained from three independent simulations of the full-length yTop I–Rpc82 complex remained consistently around ∼4.3 nm, indicating that the complex maintains structural stability throughout the simulations (Figure 6F; Supplementary Figure S11).

**FIGURE 6:**
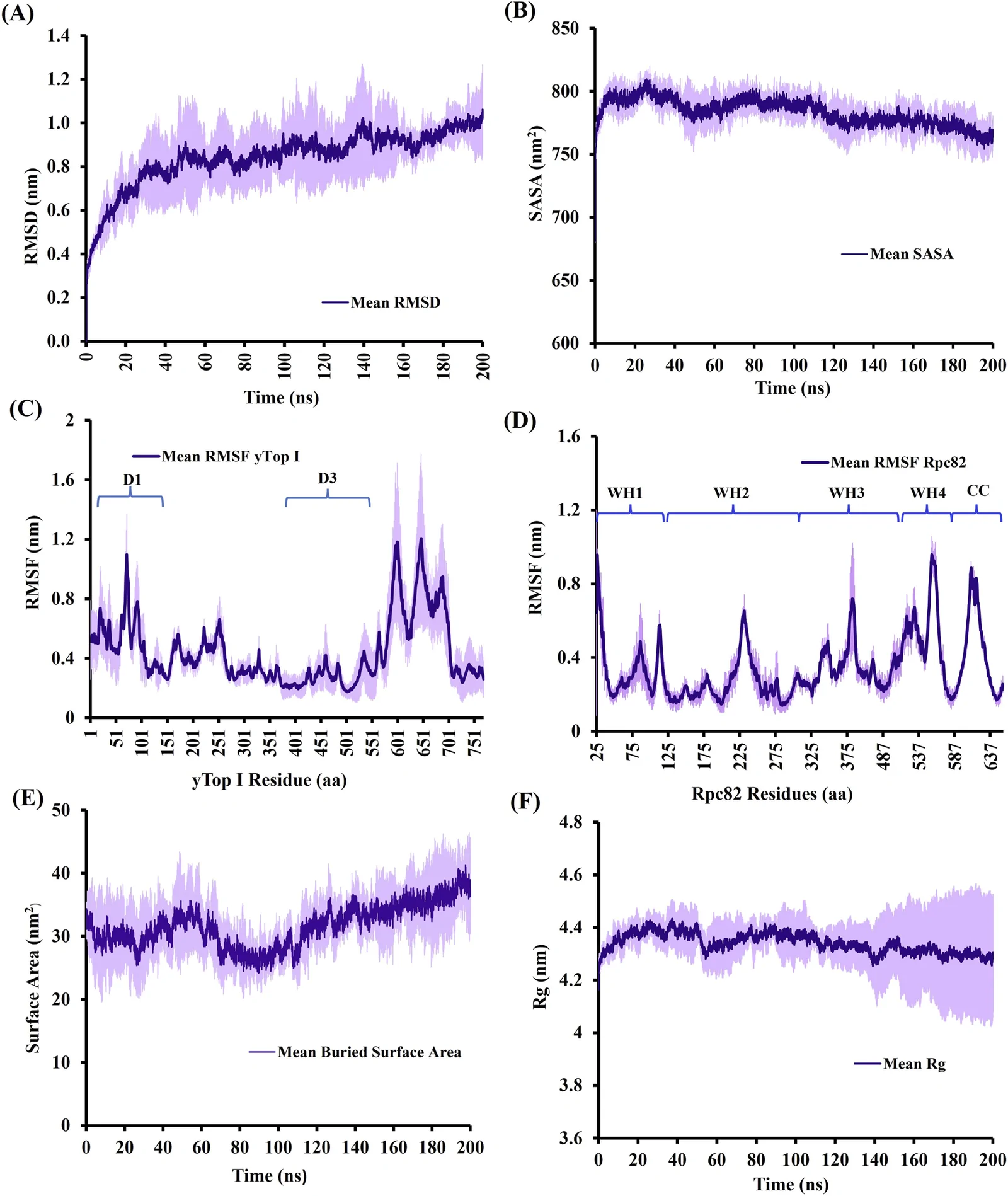
Triplicate MD simulations assessing the stability and dynamics of the *in silico*-docked yTop I–Rpc82 complex. **(A)** Mean RMSD profile of the complex obtained from three independent simulation runs. **(B)** Mean solvent-accessible surface area (SASA) profile of the complex across the triplicate simulations **(C)** Mean RMSF profile of yTop I residues obtained from the three independent simulations. **(D)** Mean RMSF profile of Rpc82 residues obtained from the three independent simulations. **(E)** Buried surface area and **(F)** Rg profiles of the complex, respectively. Dark violet curves represent the mean values across the three independent simulations, with light shaded areas indicating ±standard deviation.

### 3.8 Key interaction hotspots contributing to complex stability during simulation

In-depth analysis of the interaction surface between the two proteins during the simulation course was performed. As the three independent simulations exhibited comparable structural and dynamic behavior, one representative trajectory was selected for detailed analysis of intermolecular contacts and interacting residues to provide a representative view of the yTop I–Rpc82 interaction interface. Analysis of the 3D coordinate files extracted at 10-ns intervals from the selected trajectory using COCOMAPS2 (Table 1), together with the interacting-residue analysis (Supplementary Table S12), helped in identification of persistent interactions between specific amino acid residues of yTop I and Rpc82 throughout the simulation course. The interaction surface parameters, including buried surface area and the number and types of intermolecular contacts, exhibited a dynamic pattern, with an increase observed at 50–60 ns followed by a modest decrease at 80–90 ns. Despite these fluctuations, the interaction interface remained sufficiently stable to sustain the yTop I–Rpc82 association throughout the simulation, with the buried surface area reaching ∼40 nm^2^ (Table 1, 200ns, 3735.7 Å^2^). Amino acid residues belonging to two separate regions, namely NTD (40 -140 aa of D1 domain) and core domain (380- 560 aa of D3 domain) were observed in forming the interaction surface of yTop I. On the other hand, residues of a single interaction surface (formed by WH2 and WH3 domains) of Rpc82 (residue position: 120-300, 447,448) were revealed by this assessment. Certain residues of yTop I were revealed to be critical for maintaining this interaction throughout the simulation course, including D50, E51, E126, E127, E128 residues of the N-terminal domain (D1) as well as Y385, K400, V401, L403, E424, E427, Q486, G488, H489, Q490, Y507, and P509 residues belonging to the core-domain (D3) of the enzyme (Supplementary Table S12).

**TABLE 1:** Analysis of the yTop I–Rpc82 interaction interface using COCOMAPS2, based on the docking pose (0 ns) and simulation frames extracted at 10-ns intervals, including buried surface area (column 8), interacting surface area (column 9), and other interaction parameters.

Time frame (ns)	Total interaction	No. of Interacting Rpc82 residues	No. of Interacting Top1 residues	H-bond	Salt bridges	polar Vander waals int	Buried Surface area (Å2)	% Buried Surface Area
0	79	41	38	9	8	34	3013.9	4.2
10	62	32	30	7	6	27	2513.1	3.02
20	59	30	29	8	6	20	2446	2.94
30	67	35	32	10	6	21	2388.3	2.87
40	77	39	38	12	6	21	2882.4	3.48
50	89	45	44	6	12	27	3294	4.04
60	81	41	40	9	13	33	3059.5	3.73
70	73	38	35	6	8	16	2691.4	3.28
80	70	36	34	6	11	28	2622	3.2
90	55	30	25	6	7	20	1972.5	2.36
100	57	28	29	5	8	30	2316	2.82
110	60	32	28	5	9	30	2411.2	2.95
120	57	29	28	7	8	18	2443.1	2.98
130	60	29	31	14	7	30	2551	3.12
140	75	36	39	6	10	26	3062.6	3.76
150	77	36	41	4	8	32	3153.7	3.9
160	77	37	40	5	13	30	3184.9	3.95
170	86	41	45	9	9	34	3434.9	4.24
180	83	40	43	6	9	42	3418.5	4.27
190	81	36	45	6	10	26	3241.8	4.01
200	98	52	46	7	12	33	3735.7	4.55

Looking at the interaction surface on Rpc82 protein, several residues in the WH2 domain, namely R145, D148, E149, Y185, V192, K255, T264, D266, T269, S270, R272 and in the WH3 domain, namely I364, R368, H369, A372, E373, and H448, exhibited sustained interactions with yTop I over a prolonged period of the simulation (Supplementary Table S12). During the simulation course, persistant interaction was observed for the above-mentioned residues of both the proteins. Interestingly, during simulation, the WH2 region of Rpc82 formed a transient interaction patch involving residues K221, K224, N225, P227, R228, and L259, while the WH3 region contributed to another transient patch comprising residues like N362, D375, R377 and A450. The consistency of the interaction pattern along with the formation of additional interacting patches, indicates that the complex may undergo adaptive conformational rearrangements to preserve the physical interaction under dynamic physiological conditions.

### 3.9 Interfacial dynamics of yTop I-Rpc82 binding

For further detailed examination of the interaction surface of the yTop I–Rpc82 complex, residue-level fluctuations, structural stability, and compactness were assessed using RMSF, RMSD, and Rg analyses (Figures 7A–D). Figure 7A demonstrates the RMSF profile of yTop I, highlighting significant fluctuations across its full-length structure. The interacting regions at the N-terminal domain (NTD) and core domain (40-140 and 380-560 residues respectively) exhibited notably different fluctuation patterns compared to the full-length protein. Specifically, the interacting regions showed more constrained fluctuations, with peak amplitudes ranging between 0.4 and 1.0 nm, with lowest value at D3 indicating potential stabilization of these critical interaction sites. Figure 7B illustrates the RMSF characteristics of Rpc82, with the full-length protein and its interacting regions (120-300 residues) displaying distinct fluctuation profiles. The interacting region demonstrated more pronounced structural dynamics, with fluctuation peaks reaching up to 0.8 nm, suggesting potential conformational flexibility critical for protein-protein interactions. The RMSD analysis (Figure 7C) tracked the structural stability of the proteins over the 200 ns simulation time. The whole complex and the interacting regions of both Rpc82 and yTop I exhibited progressively stabilizing behavior, with RMSD values converging around 0.8–0.9 nm. Notably, the interacting regions of yTop I and Rpc82 maintained lower RMSD values of approximately 0.6 nm for most of the simulation, indicating greater stability at the interaction interface and supporting a consistent protein–protein interaction between yTop I and Rpc82. The interaction surfaces for yTop I and Rpc82 also exhibited comparable compactness, with Rg values of ∼3.4 nm and ∼2.2 nm respectively (Figure 7D). Very little deviation in Rg values (around 1 nm) observed throughout the simulation trajectory indicates stability of the complex. The average SASA values of individual interacting regions were found to be ∼320 nm^2^ for yTop I and ∼280 nm^2^ for Rpc82. These values are considerably lower than observed values for the full protein complex (∼800 nm^2^), and this trend was maintained throughout the simulation period (Supplementary Figure S12). Residue-wise changes in the accessible surface area were also measured for the selected residues of both the protein and the result indicated the sustained interaction between the two proteins throughout the duration of simulation. The residue-specific SASA values of the interacting surface were found to be placed around 100 nm^2^ for Rpc82 protein and ∼220–230 nm^2^ for yTopI protein, indicating reduced solvent exposure upon complex formation (Supplementary Figure S12).

**FIGURE 7:**
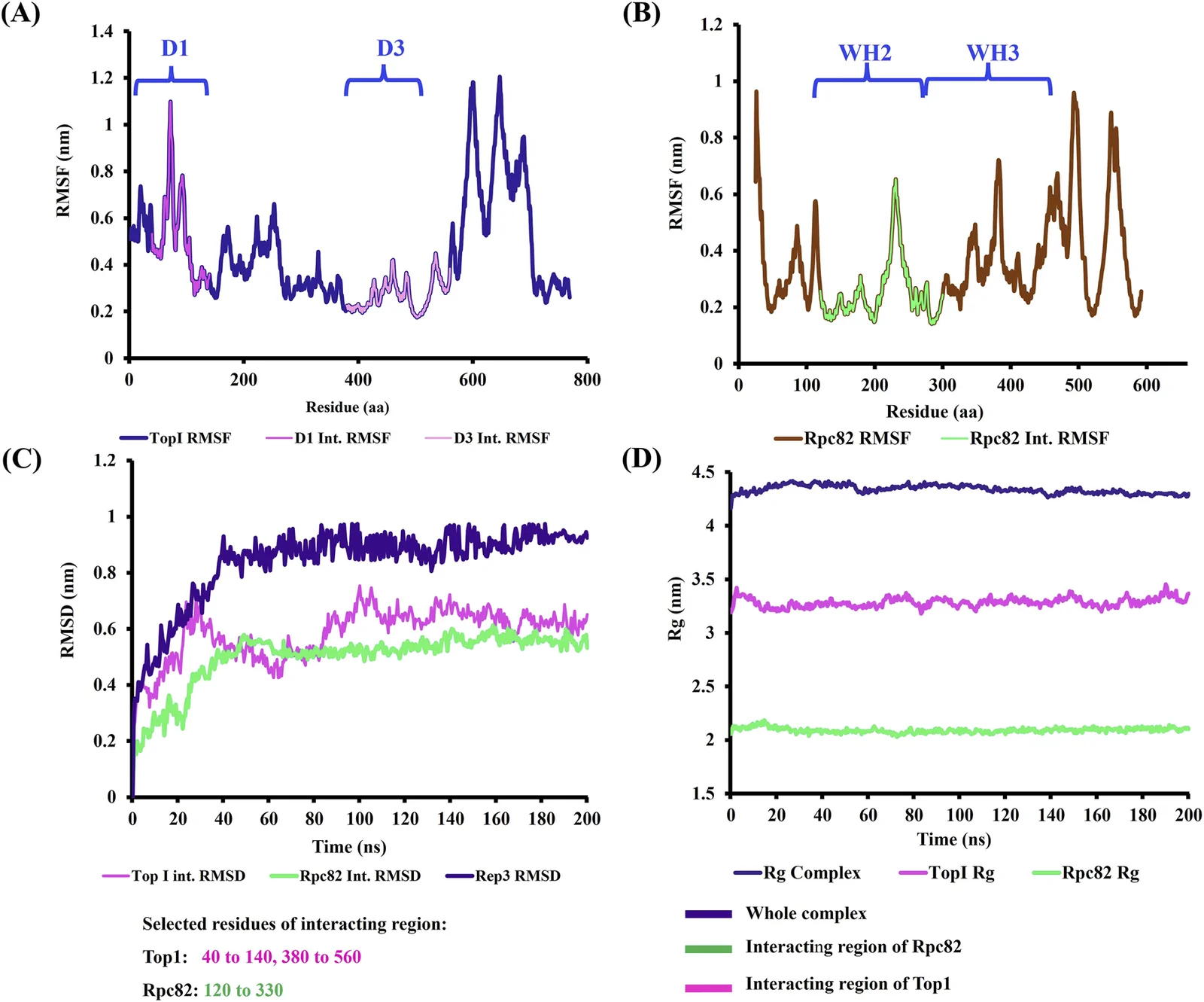
Molecular Dynamics based evaluation of interaction between Topoisomerase I (yTop I) and RNA polymerase III subunit Rpc82 Interaction **(A)** RMSF profile of full-length yTop I highlighting residue-level fluctuations, particularly at interaction regions. **(B)** RMSF analysis of Rpc82 showing domain-specific flexibility **(C)** RMSD profiles of the interacting interfaces of yTop I and Rpc82 compared with the whole-complex RMSD over 200 ns, assessing the stability of the interaction regions. **(D)** Rg profiles of the whole yTop I–Rpc82 complex and its interacting interfaces on yTop I and Rpc82, demonstrating compactness and conformational stability throughout the 200 ns simulation.

MM-PBSA analysis revealed an overall favorable energetic profile for the yTop I–Rpc82 complex (Figure 8). The yTop I–Rpc82 complex exhibited an overall decrease in system energy throughout the 200 ns simulation, with the energy fluctuating around a relatively stable range after the initial equilibration phase, and remained around −37,300 kcal/mol toward the later stages of the simulation, indicating energetic stabilization of the simulated complex (Figure 8A). Similarly, the binding free energy of the interacting patch (ΔG_bind_) showed an overall shift toward more negative values, despite considerable temporal fluctuations and reached to −300 to −310 kcal/mol towards the end of simulation, indicating energetically favorable and progressively stabilized interactions between yTop I and Rpc82 (Figure 8B).

**FIGURE 8:**
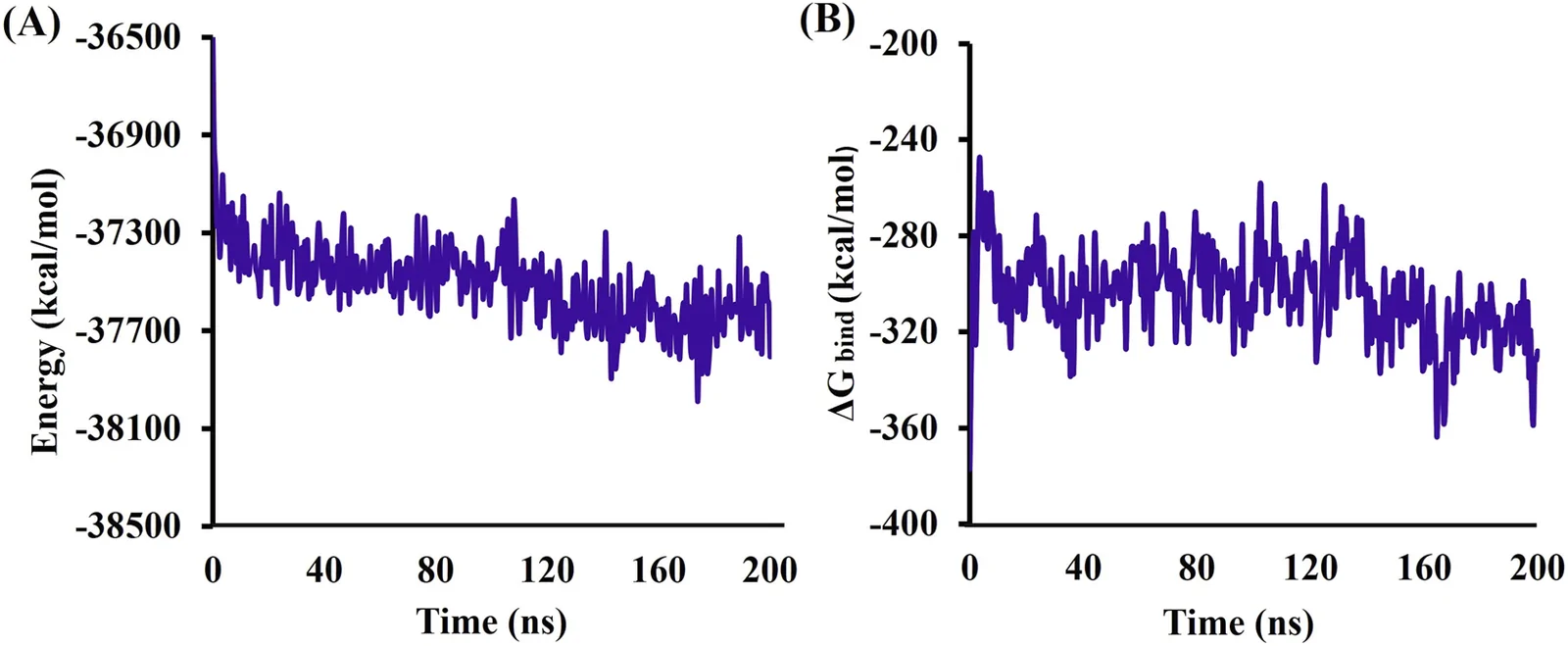
yTop I- Rpc82 complex energy profile and MM-PBSA binding free-energy analysis of the yTop I–Rpc82 complex **(A)** Time-resolved energy plot of the yTop I–Rpc82 complex over the 200 ns simulation trajectory. **(B)** MM-PBSA–based binding free energy calculation of the interaction interface, confirming the energetically favorable nature of the complex.

## 4 Discussion

In this study, a full-length structural model of yeast Topoisomerase I (yTop I) was generated using a template-based modeling approach, which enabled inclusion of regions, particularly the N-terminal domain (D1) that are often predicted as disordered in AlphaFold and homology-based predictions. We believe that this alternative approach may offer a useful strategy for exploring the structural organization of large and highly flexible proteins. However, further experimental validation would be required to confirm its accuracy.

Multiple advanced modeling tools were employed to maximize the number of initial models of individual domains in an unbiased manner. The final selection of representative models for each segment was based on extensive validation using ERRAT, Verify3D, and Ramachandran plot analyses, ensuring successful assembly of individual domains of yTop I into a reliable full-length structure. On the basis of rigorous comparative assessment across multiple structural quality parameters and benchmarking against available high-resolution protein structures, a qualitative evaluation of the selected domain models and the full-length yTop I was achieved. Additionally, comparative assessment of the selected full-length yTop I model with structures generated using GalaxyWEB, Protenix, and AlphaFold further supported the consistency of the chosen model, suggesting its potential reliability within the *in silico* framework. Collective comparisons support the robustness and reliability of the selected full-length yTop I model to proceed for further downstream interaction studies.

The final full-length model was assembled using MODELLER (Eswar et al., 2006), followed by further structural validation. The resulting structure suggests some degree of flexibilities at the N-terminal domain and linker domain regions, whereas the DNA-binding domain, core, and C-terminal domains appear to have stable modular organization. This observation is broadly consistent with the established functional roles of these domains in the catalytic cycle of the enzyme. Structural superimposition of the predicted yTop I model with the AlphaFold v6-predicted structure and the human Top I crystal structure (PDB ID: 1K4T) revealed relatively small deviations and good overall alignment, particularly within conserved regions surrounding the active site and DNA-binding interface.

As topoisomerases are known to play critical role in DNA transcription, we further explored molecular interaction between yeast Topoisomerase I with RNA pol III subunit Rpc82 as they were shown to interact previously in high-throughput protein-protein interaction studies (Chymkowitch et al., 2017; Ho et al., 2002). Pol III-transcribed genes are generally short and undergo rapid rounds of transcription reinitiation, resulting in frequent recycling of RNA polymerase III. Such intense transcriptional activity can lead to the accumulation of transcription-induced DNA supercoiling and torsional stress if not properly resolved (Dieci et al., 2013). In budding yeast, where most tRNA genes are actively occupied and heavily transcribed under nutrient-rich growth conditions, the need for efficient management of local DNA topology is critical (Dieci et al., 2013). In this context, physical association between RNA polymerase III and Topoisomerase I may help relieve transcription-associated torsional strain and support sustained, high-level Pol III transcription in budding yeast.

The molecular docking results between full-length yTop I and Rpc82 protein indicated the primary involvement of N-terminal domain (D1) as well as residues belonging to the core-domain (D3) of yTop I. Interestingly, Top I-NTD is often implicated in regulatory interactions. It has been shown to be involved in interaction with RNA pol II, Sir2 (D’Alfonso et al., 2016). Thus, the D1 domain may serve as a flexible docking platform with surface-exposed motifs or conserved patches serving as hotspots for interaction by different partners of Top I like Rpc82, depending on cellular context. D3 core domain of yTop I has been previously shown to interact with Tof1, a component of the fork protection complex. yTop I-Tof1 interaction plays a crucial role in replication fork pausing at programmed pause sites like the ribosomal DNA (rDNA) replication fork barrier (Park and Sternglanz, 1999). Although our initial docking analysis revealed two distinct binding surfaces on Rpc82, the binding interface comprising the WH2–WH3 domains was prioritized for further analysis as this interaction site is unlikely to interfere with RNA polymerase III function, being spatially distinct from the regions implicated in RNA polymerase III holoenzyme assembly. The association between the two proteins could potentially position yTop I near actively transcribing loci, where it may contribute to the resolution of transcription-induced DNA supercoiling. Topoisomerase I plays a critical role in resolving transcription-induced DNA supercoiling, and its proximity to RNA polymerase III could enable rapid relaxation of torsional stress, thereby supporting uninterrupted elongation. Moreover, previous studies focusing on RNA polymerase - Topoisomerase I physical interaction till date, demonstrated a positive impact of this interaction in maintaining transcriptional fidelity and genome stability. Notably, Pol III transcription factors in human, TFIIIC has been shown to bind human Topo I (with PC4), stimulating Pol III reinitiation (Z. Wang and Roeder, 1998b). The second predicted binding surface on Rpc82 (formed by WH1-WH4, CC domain) was not considered for further detailed analysis, as this region is involved in RNA polymerase III holoenzyme assembly, wherein interactions with other core subunits would likely lead to steric hindrance.

Comparative analysis of the yTop I-Rpc82 complex with known heterodimers showed broadly comparable trends in binding energy, energy/surface area profile, consistent with established benchmark protein complexes and supporting the structural and energetic feasibility of the interaction under physiological conditions. MD simulations indicated relatively stable interaction profiles over the simulated timeframe of 200 ns, with some residues showing persistent contacts at the interface. RMSD trajectories of the model suggest that the complex retains structural integrity and dynamic stability under simulated physiological conditions. To assess the statistical confidence of the simulation observations of yTop I–Rpc82 complex under a cell-like environment, triplicate MD simulations were performed. The average deviation (RMSD) (Figure 6A) and SASA profiles (Figure 6B) exhibited a consistent pattern across all replicates, in agreement with the initial observations. The RMSD values remained predominantly within the 0.7–0.9 nm range for most of the simulation period, indicating overall structural stability of the complex. The yTop I–Rpc82 complex also maintained a nearly constant accessible surface area of approximately ∼800 nm^2^ across repeated simulation cycles, further supporting structural consistency. The predicted docking pose thus emerged as energetically favourable, structurally stable and represents a biologically plausible interaction between the two proteins. Rpc82 subunit, an essential component of RNA polymerase III holoenzyme with a specified role during transcription initiation (Khoo et al., 2018) may contribute to the recruitment of yTop I to actively transcribing loci.

SASA and Rg values indicated the structural compactness of the overall protein complex as well as the stability of the interaction interface. Detailed analysis of the simulation trajectories revealed that several residue pairs maintained intermolecular interactions for extended periods, indicating the formation of a stable protein–protein binding interface. Such an association could facilitate local resolution of transcription-induced DNA supercoiling and promote transcriptional fidelity, although this remains to be experimentally validated. MD simulation studies indicated relatively stable contacts at the predicted interface and highlighted a few potential interaction hotspots. Several interesting features emerged from the interaction analyses. A cluster of charged residues within the D1 region of yTop I, including D42, E43, E45, D50, E51, K123, K125, E126, and E127, remained persistently involved in interactions with Rpc82 despite the relatively higher RMSF observed in the intervening region spanning residues 60–100. Notably, residues 489–509 of yTop I showed more frequent involvement in the interaction during the later phase of the simulation compared with the early phase (Supplementary Table S12). Similarly, residues within the WH3 region of Rpc82, including R368, H369, A372, R377, H448, and A450, became more prominently involved in establishing the yTop I–Rpc82 association after 120 ns and persisted until the end of the 200-ns trajectory (Supplementary Table S12). Overall these findings provide preliminary but crucial insights into a plausible RNA Pol III–Topoisomerase I interaction and offer a basis for future experimental investigation of its functional relevance.

## 5 Conclusion

Topoisomerase I in yeast plays a critical role in maintaining DNA topology during essential cellular processes and contributes to genome stability. However, the absence of a well-defined full-length structure has limited structure-based interactome studies and hindered detailed insights into its functional partnerships. This study presents a computationally derived structural model of full-length yTop I using a domain-based modeling strategy, evaluated through multiple validation metrics, offering a preliminary framework for future structural investigations.

Using the model, we explored a potential interaction between yTop I and the Rpc82 subunit of RNA polymerase III. Molecular docking, MD simulations, and binding free energy analyses suggest a structurally stable and energetically favorable interaction interface and identify candidate residues that may contribute to binding. However, these findings are derived entirely from computational approaches and therefore represent a testable structural hypothesis rather than direct evidence of a physical or functional interaction. Moreover, the proposed roles of this interaction in transcriptional regulation or genome maintenance require future experimental validation.

Despite these limitations, the identification of putative interaction hotspots provides a useful starting point for future experimental studies, including targeted mutagenesis and biochemical validation. This work offers a preliminary structural framework that may inform and guide future studies aimed at exploring the physiological relevance of the Top I–Rpc82 interaction and deepening our understanding of the dynamic interplay between transcription and DNA topology in yeast cells. Given the evolutionary conservation of both proteins, it is possible that aspects of this interaction may extend to other eukaryotic systems, including humans. However, any potential relevance of a Top I–RNA polymerase III interaction in higher eukaryotes and its implications in disease contexts such as cancer, will require substantial experimental investigation.

## Data Availability

All data generated and analyzed during this study, as well as the computational resources utilized, are publicly accessible. The identification of Topoisomerase I interactors was performed through comprehensive searches in PubMed (https://pubmed.ncbi.nlm.nih.gov/), BioGRID (http://thebiogrid.org/), and STRING (https://string-db.org/) databases. Domain architecture information for yeast Topoisomerase I (yTop I) was obtained from the InterPro server (https://www.ebi.ac.uk/interpro/). Structural models of yTop I domains were rigorously predicted using a variety of algorithmic means like Ab initio, DeepFold and RoseTTAFold through the Zhang Lab server (https://zhanggroup.org/research/#StructurePrediction) and the Robetta server (https://robetta.bakerlab.org/). The crystallographic structure of yeast Topoisomerase I was retrieved from the RCSB Protein Data Bank (PDB ID: 1OIS; https://www.rcsb.org/structure/1OIS). Structural quality of the predicted models were evaluated by ERRAT, Verify3D, Ramachandran plot parameters utilising the SAVES v6.1 (https://saves.mbi.ucla.edu/) server. Assembly of individual yTop I domains was performed by the MODELLER software package (https://salilab.org/modeller/). Molecular docking analyses were conducted using three independent servers, ensuring the robustness of our findings: ClusPro (https://cluspro.org/), HDOCK (http://hdock.phys.hust.edu.cn/), and HADDOCK 2.4 (https://rascar.science.uu.nl/haddock2.4/). Interaction parameters including binding energy, interaction surface area etc. were estimated using PDBePISA (https://www.ebi.ac.uk/pdbe/pisa/) and PRODIGY (https://rascar.science.uu.nl/prodigy/) servers. For preparing standard graphs of binding energy and energy/surface area of published homodimers, protein information were collected from RCSB-PDB database (https://www.rcsb.org/structure/), interacting surface area were measured using PDBsum (https://www.ebi.ac.uk/thornton-srv/databases/pdbsum/Generate.html) program and binding energy were evaluated by PRODIGY (https://rascar.science.uu.nl/prodigy/) server. Structural visualization and superimposition of protein structures were achieved by utilizing the Biovia Discovery Studio 2024 free version (https://discover.3ds.com/discovery-studio-visualizer-download), UCSF Chimera X 1.19 (https://www.cgl.ucsf.edu/chimerax/cgi-bin/secure/chimerax-get.py?file=1.9/windows/ChimeraX-1.9.exe) and SuperPose program (https://superpose.wishartlab.com/). Binding free energy calculations were performed using the gmx_MMPBSA package (https://github.com/Valdes-Tresanco-MS/gmx_MMPBSA). Raw data files, including model prediction, docking results, MD simulation using installed Gromacs (https://www.gromacs.org/) program and interaction analyses, are available from the corresponding author and archived at Zenodo (https://zenodo.org/records/21863991).
