# Supplementary material for: Exploring molecular interactions between yeast topoisomerase I and RNA polymerase III subunit, Rpc82 using an in-silico approach
Source: Front Bioinform. 2026 Oct 9;6:1897635. doi: 10.3389/fbinf.2026.1897635 (PMC13690749; doi:10.3389/fbinf.2026.1897635)
Supplement: Supplementary file 1 [file DataSheet1.pdf]

SUPPORTING INFORMATION

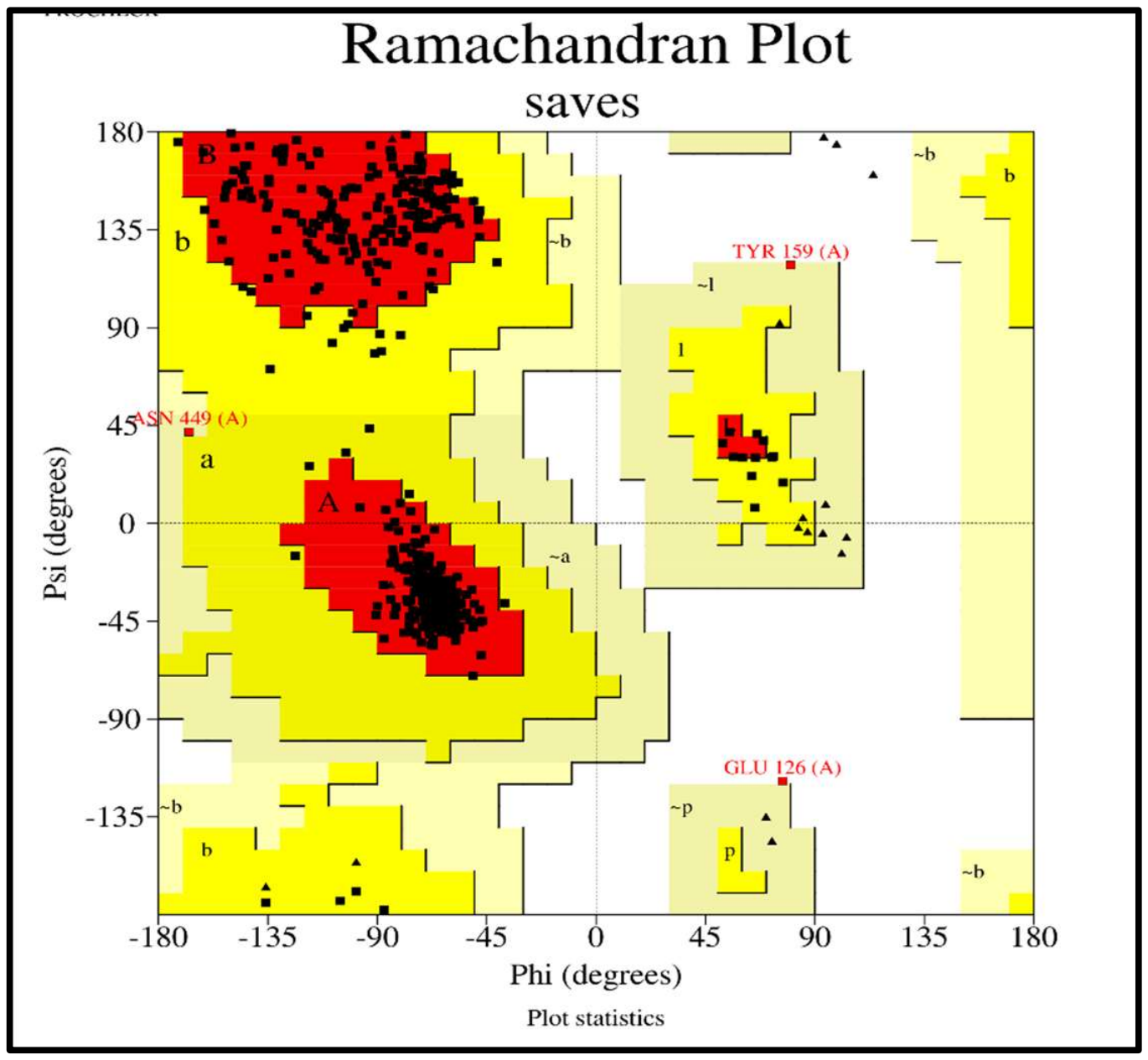

Supplementary Figure S1: Ramachandran plot for the predicted model of the full-length Topoisomerase-I from budding yeast, indicating the presence of maximum residues at the favoured regions.

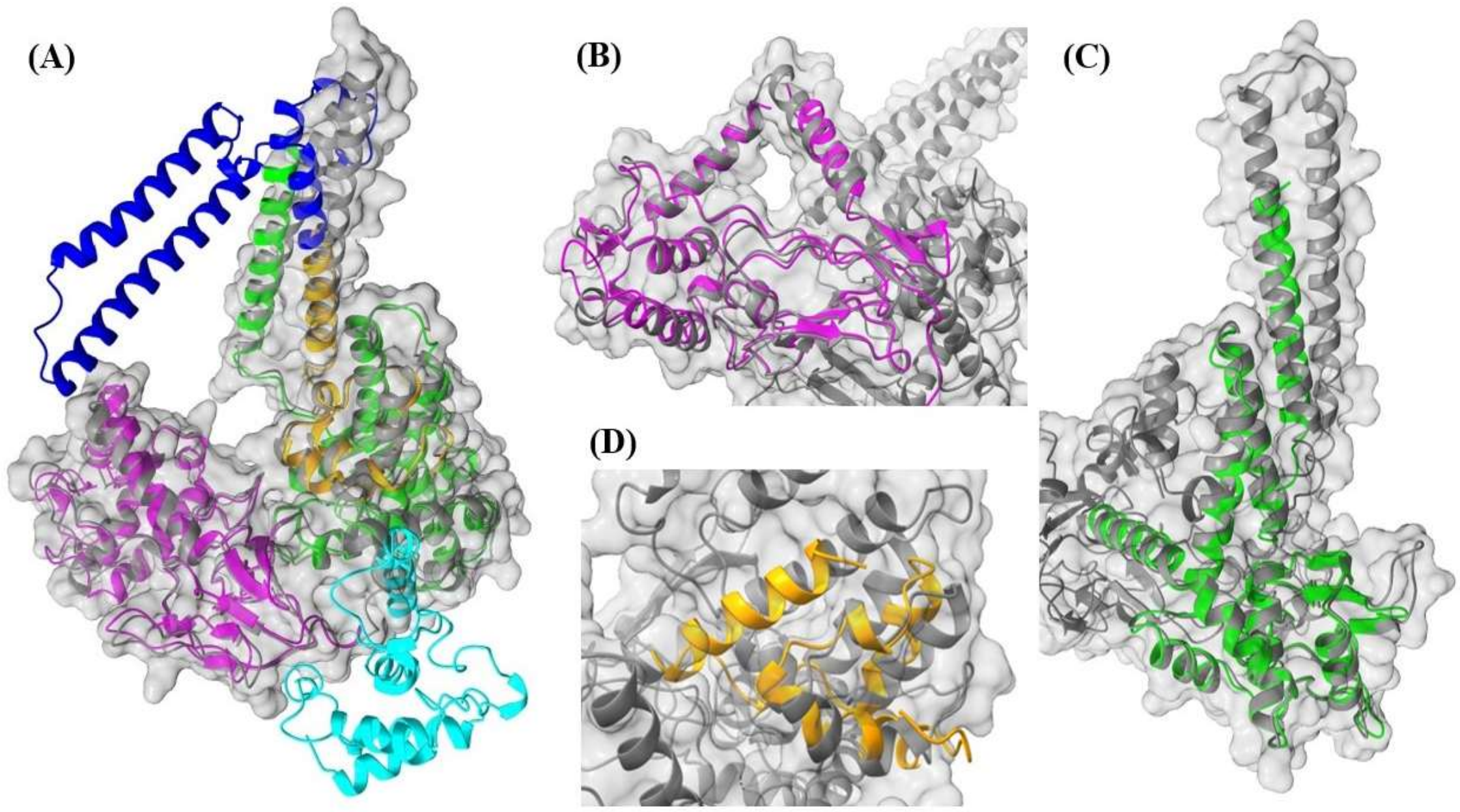

Supplementary Figure S2: Superposition of the predicted full-length yeast Topoisomerase I (yTop I) model with the human Topoisomerase I crystal structure (PDB ID: 1K4T; grey). A- Domain-wise superposition of yTop I, with D1–D5 shown in cyan, magenta, green, blue, and orange, respectively (RMSD = 1.021 Å). B- Enlarged view of superimposed region of D2 (magenta) of yTop I on human Top I Magnified view of yTopI D5 region (orange) superimposed on hTopI. C- superimposed region of D3 (green) of yTop I on human Top I (grey). D-Magnified view of yTopI D5 region (orange) superimposed on hTopI (grey).

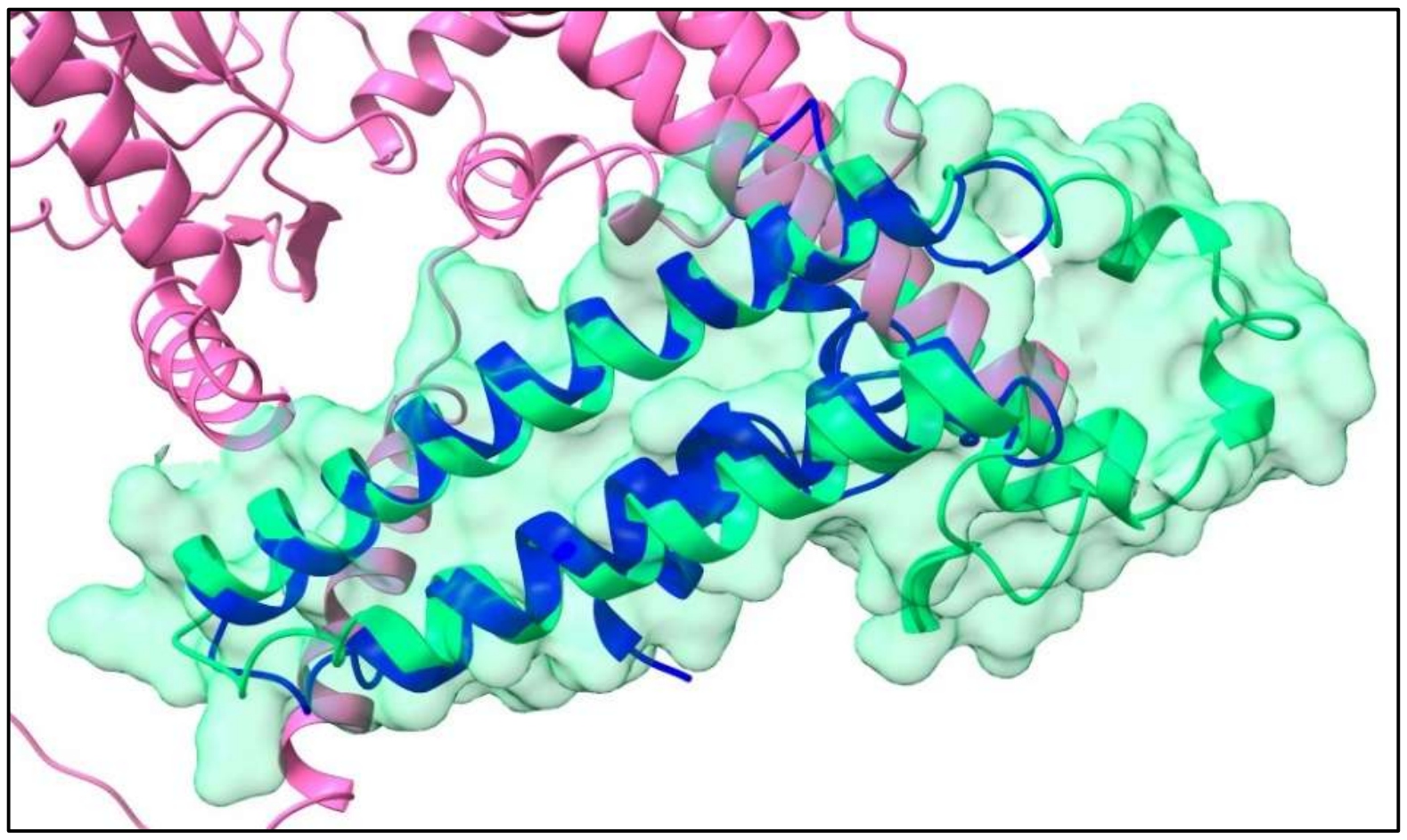

Supplementary Figure S3: Superposition of the predicted D4 linker region of yeast Topoisomerase I (blue) onto the corresponding region (residues 590–698; green) in the AlphaFold-predicted model for full-length yeast Topoisomerase I (pink).

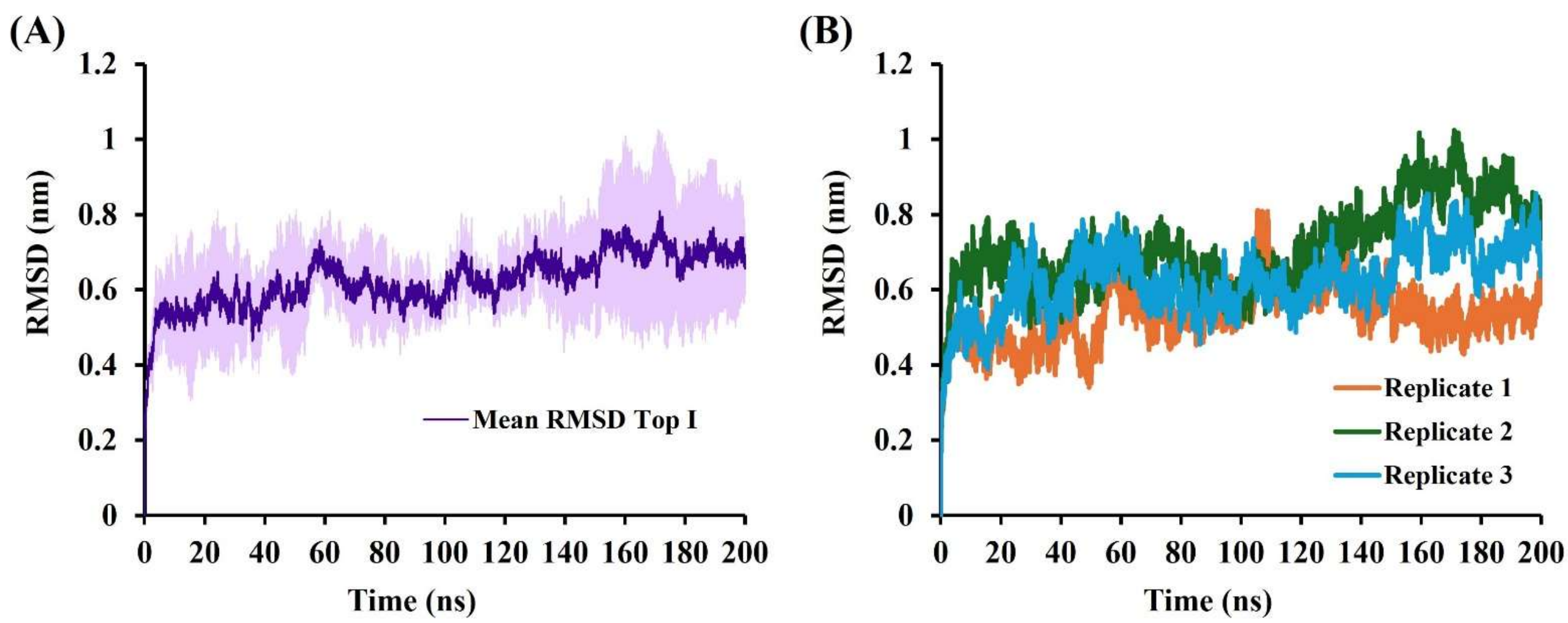

Supplementary Figure S4: **RMSD plots from three independent MD simulation runs of the yTopI model. (A) Mean RMSD across the triplicate runs. (B) RMSD profiles of the individual runs.**

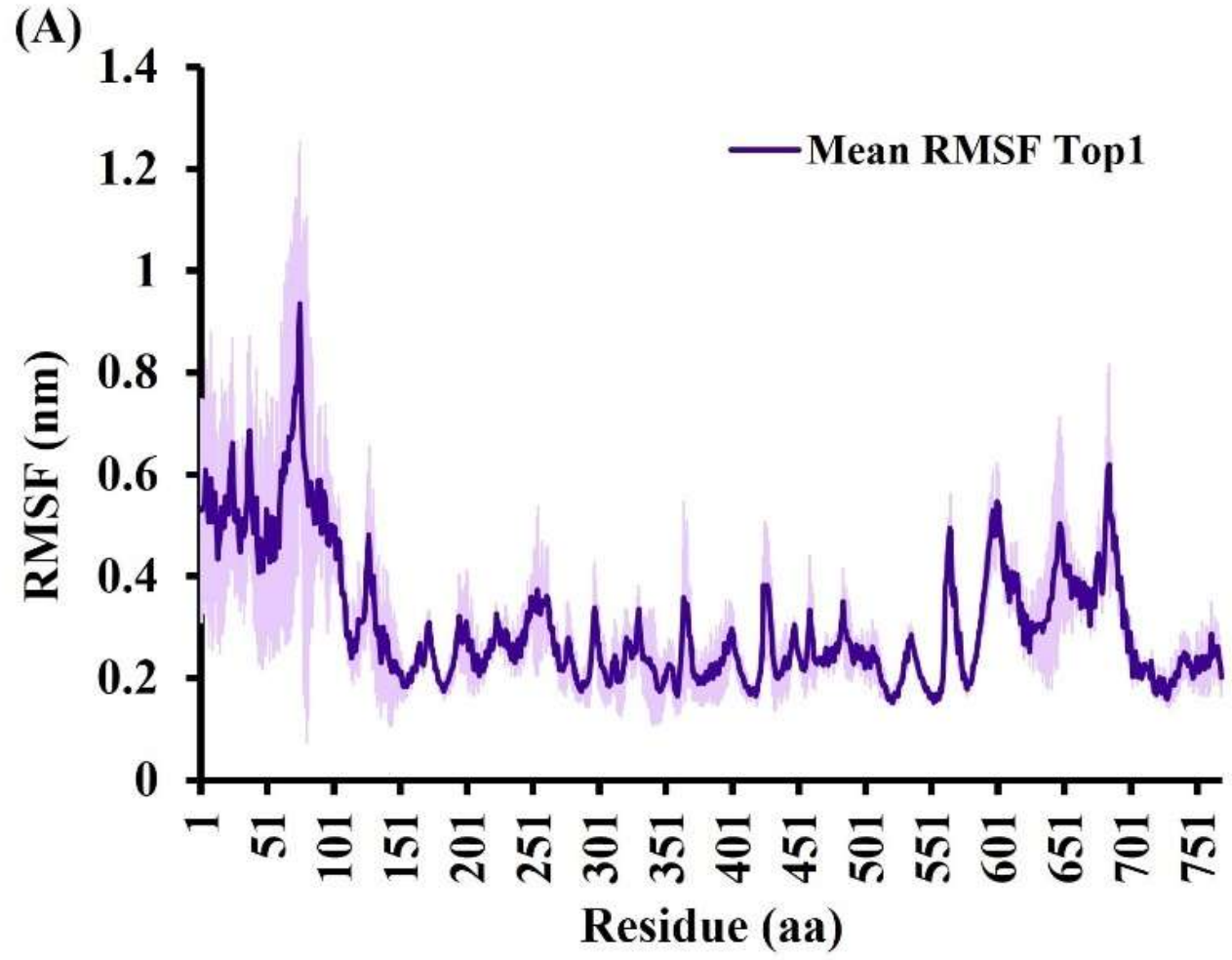

Supplementary Figure S5: RMSF plots from three independent MD simulation runs of the yTopI model. (A) Mean RMSF profile across the triplicate runs. (B) RMSF profiles of the individual runs.

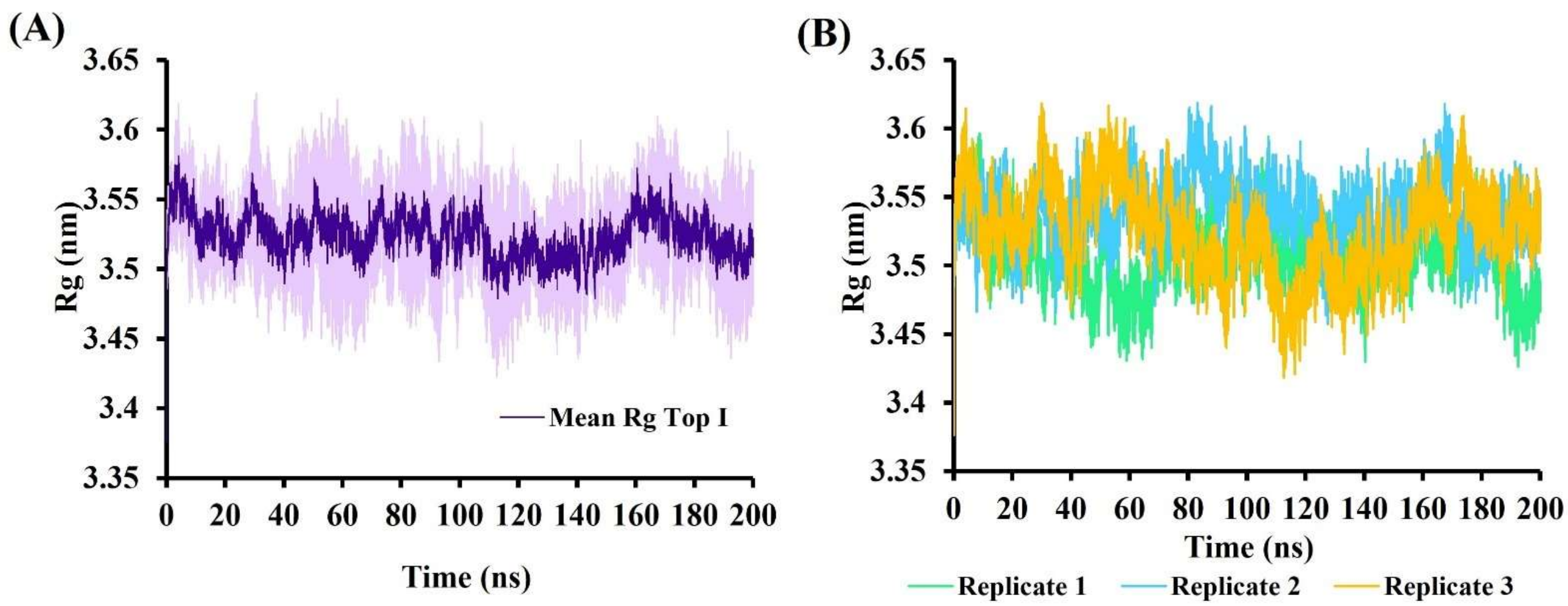

Supplementary Figure S6: Rg plots from three independent MD simulation runs of the yTopI model. (A) Mean Rg profile across the triplicate runs. (B) Rg profiles of the individual runs.

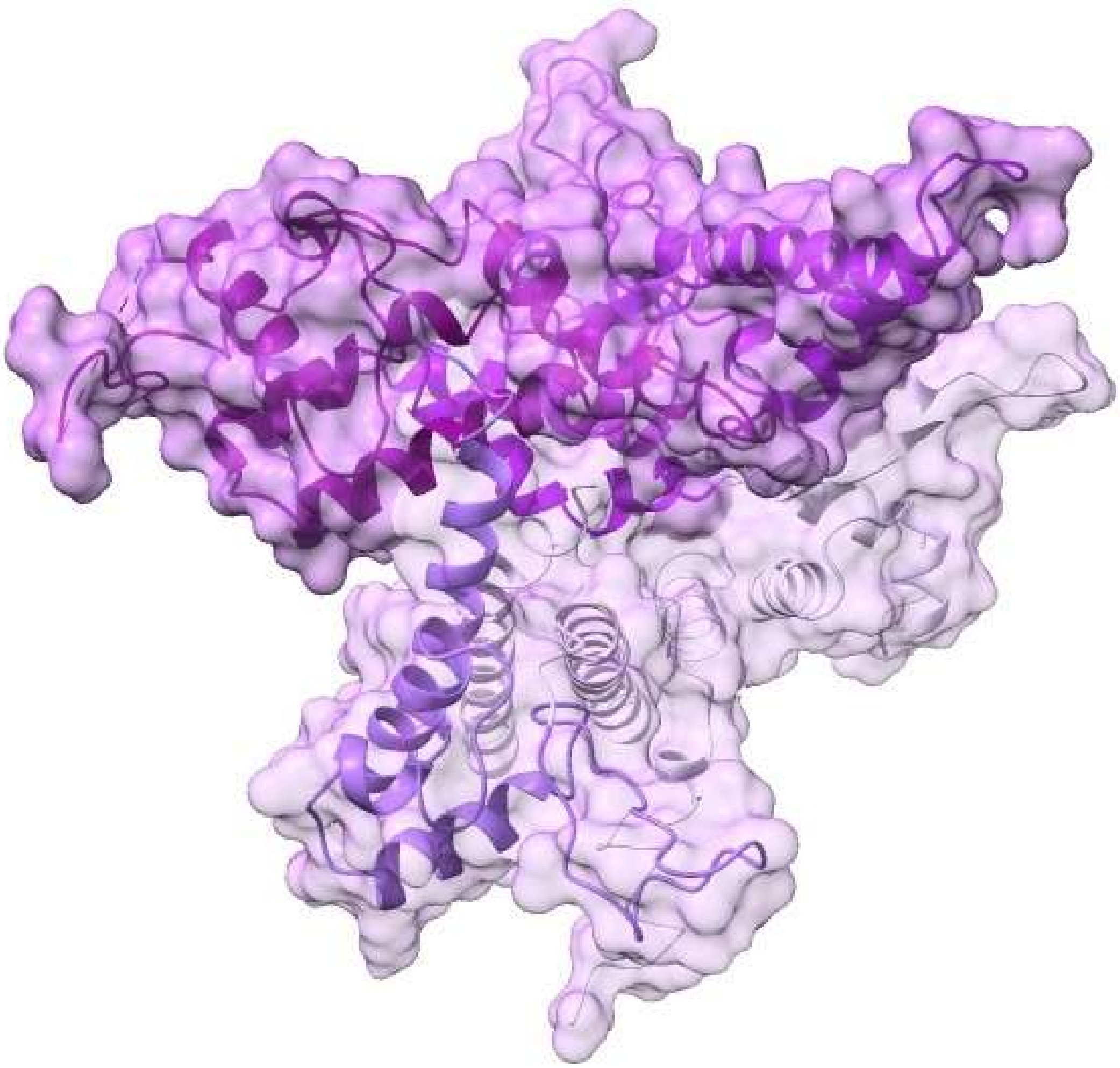

Supplementary Figure S7: Distinct molecular surfaces of Rpc82, with the WH1–WH4–CC region shown in light violet and the WH2–WH3 region in dark violet.

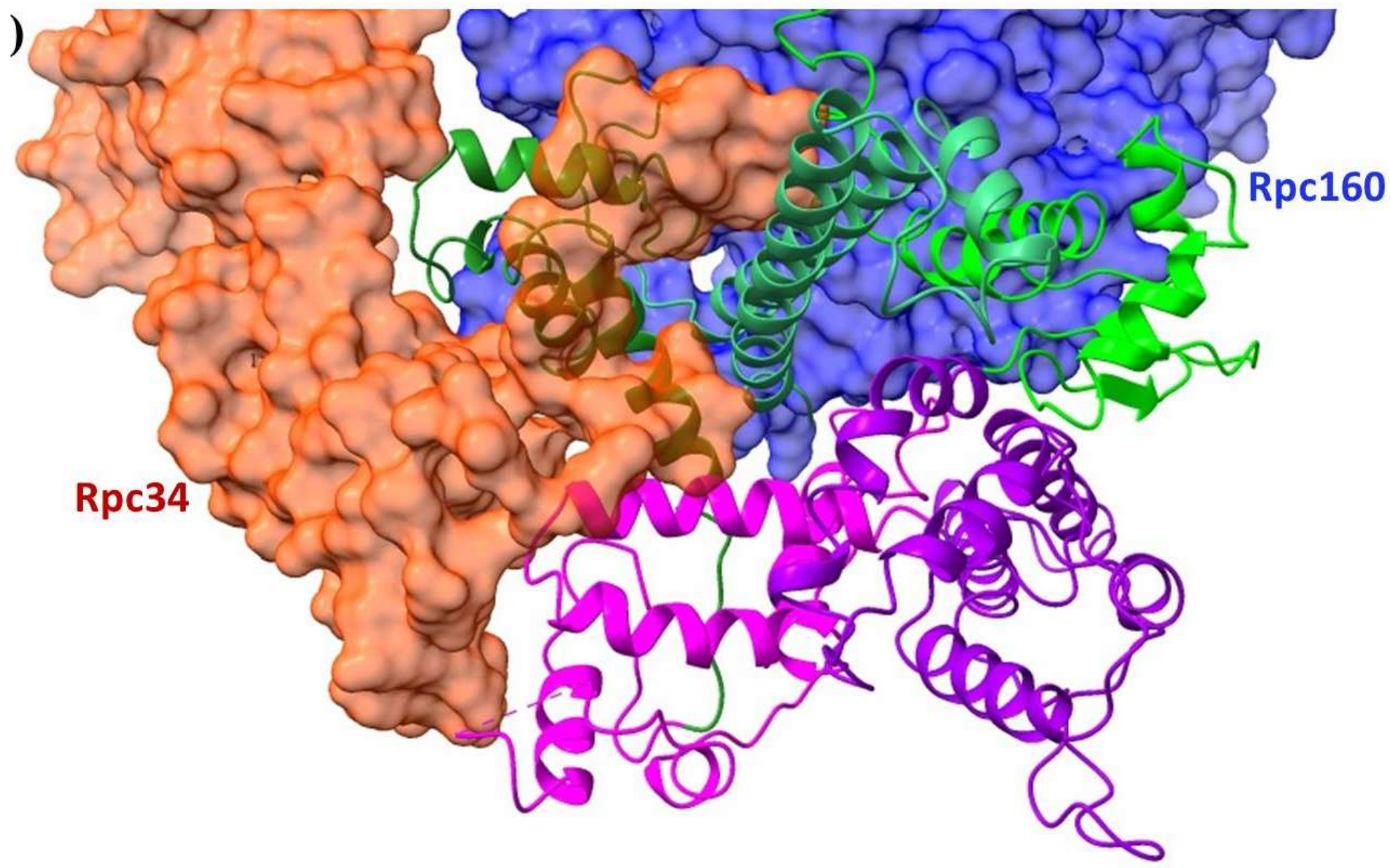

Supplementary Figure S8: Structural assembly analysis of RNA Pol III holoenzyme (PDB ID: 6EU0) revealed that Rpc34 (red) and Rpc 160 (Blue) interacts with the WH1-WH4-CC made surface (green) of Rpc82 protein and the WH2 (Violet) and WH3 (Purple) are unoccupied by any subunits.

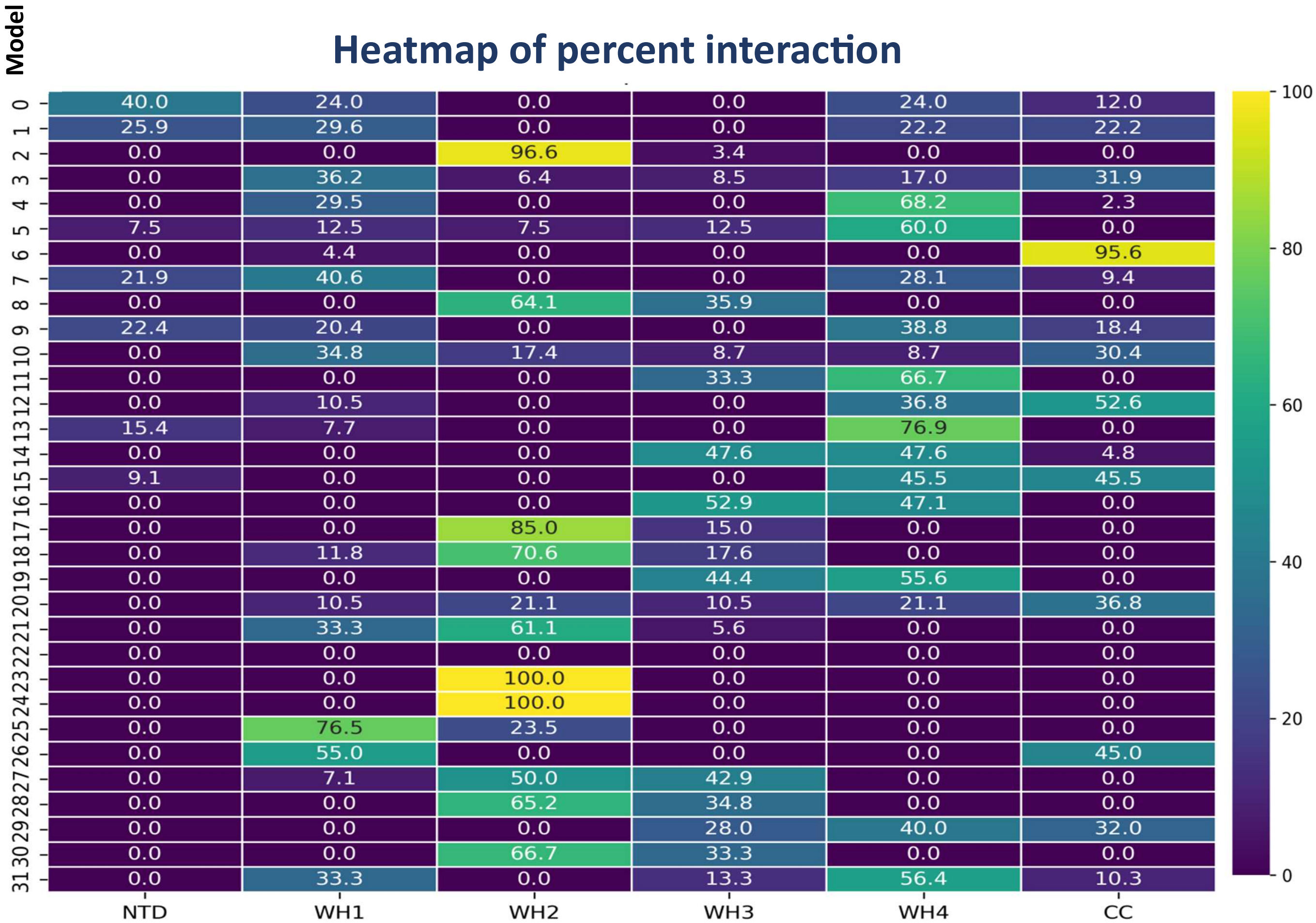

Supplementary Figure S9: Heatmap analysis to identify which domains of Rpc82 interact preferentially with different docking models of yTop I, using color gradients to determine the interaction intensity. It provides percent interaction values across different domains of Rpc82 (NTD, WH1-WH4 and CC) for the different docking models involving full-length yTop I. Interaction data for Rpc82's NTD, WH domains (WH1, WH2, WH3, WH4) and the coiled-coil (CC) domain with full-length yTop I from docking simulations were gathered and structured in a format that includes domains as columns and docking models as rows, with the cell values representing the percentage of interaction observed in the model. Data from 30 models have been arranged sequentially with 10 best models each from ClusPro, HDOCK and HADDOCK server respectively (0-29), model 30 for Rpc82-Rpc34 interaction and model 31 row indicating Rpc82-Rpc160 interaction profile.

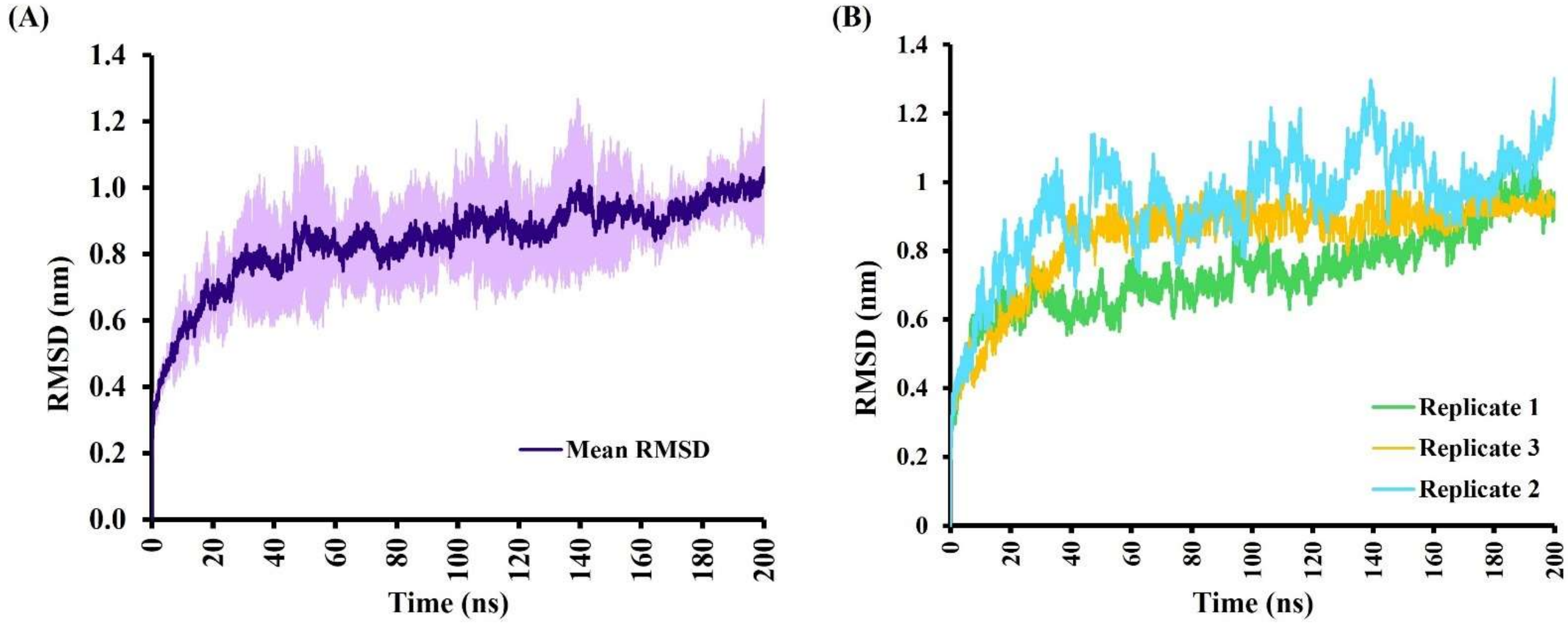

Supplementary Figure S10: RMSD plots from three independent MD simulation runs of the yTopI–Rpc82 docking complex. (A) Mean RMSD across the triplicate runs. (B) RMSD profiles of the individual runs.

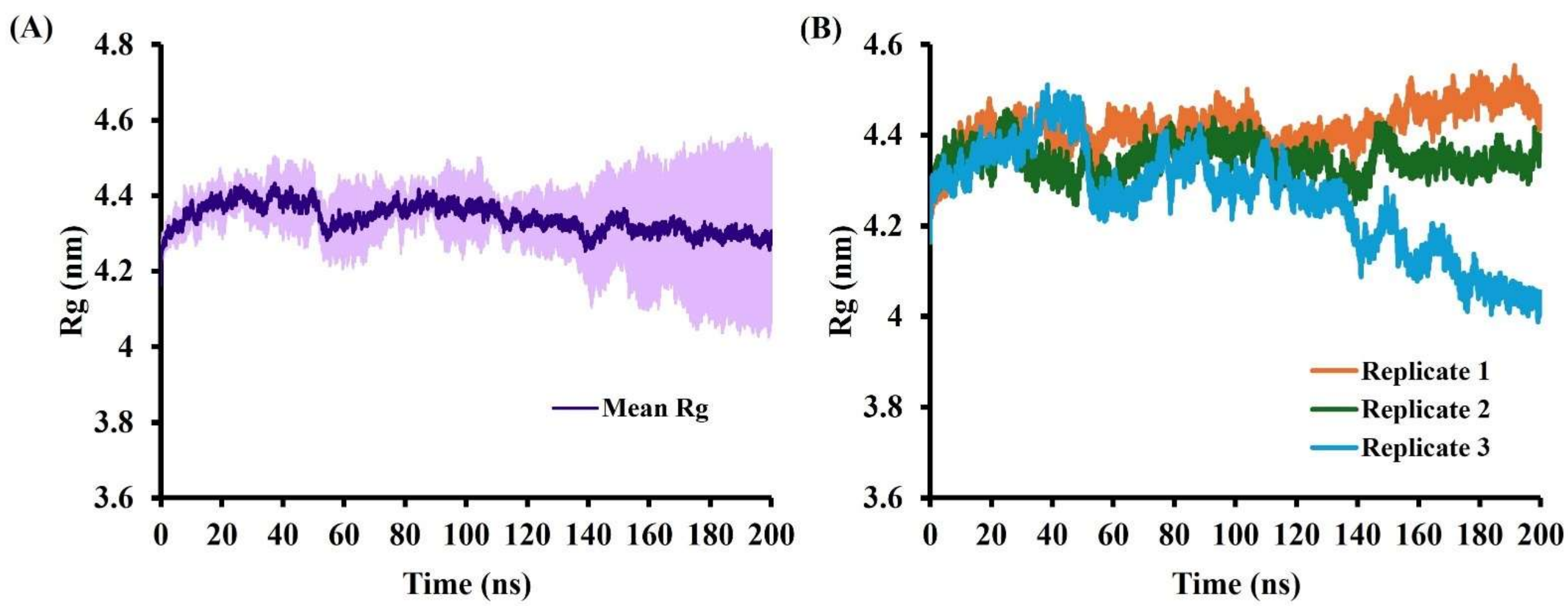

Supplementary Figure S11: Rg plots from three independent MD simulation runs of the yTopI–Rpc82 docking complex. (A) Mean Rg across the triplicate runs. (B) Rg profiles of the individual runs.

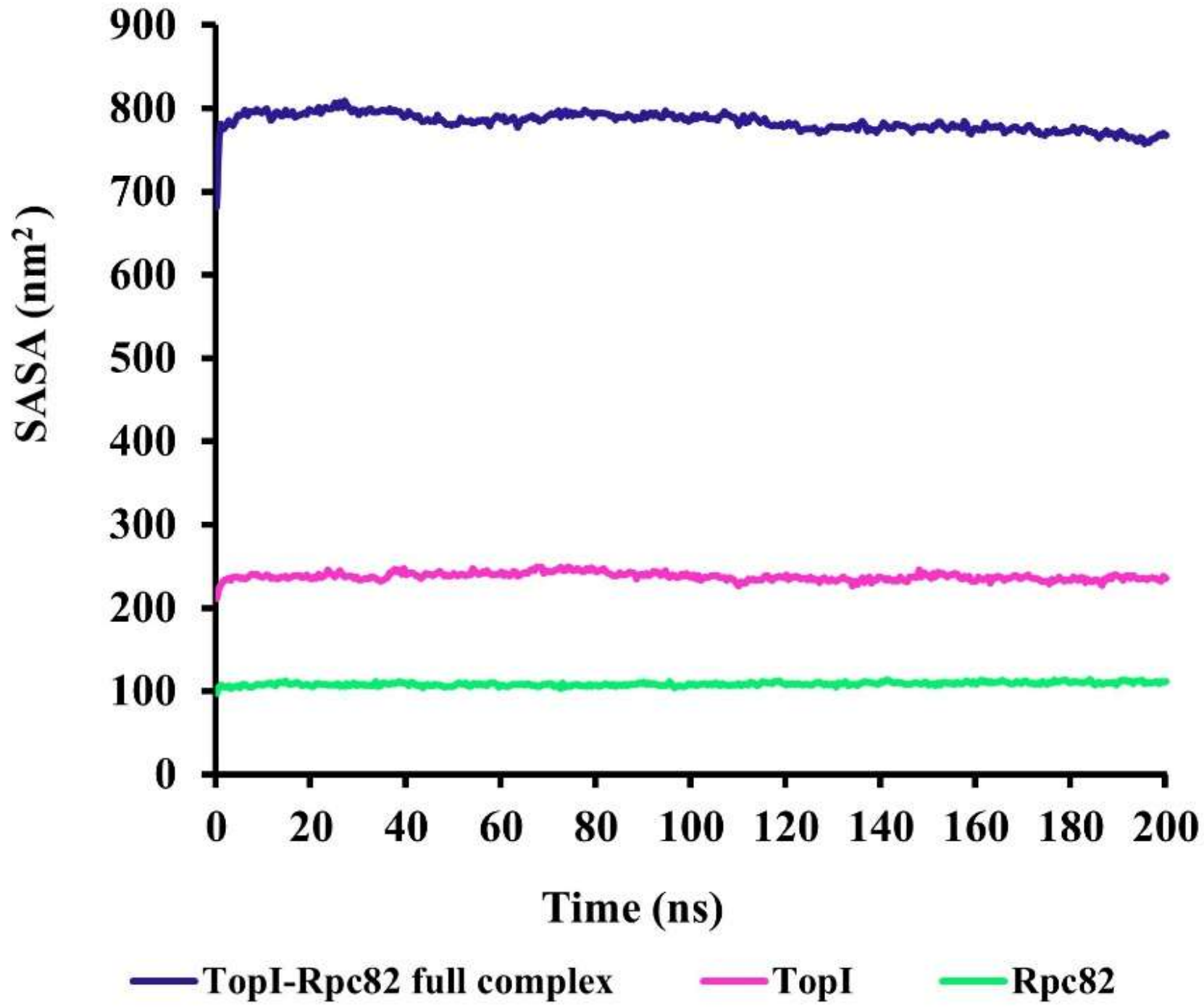

Supplementary Figure S12: SASA plot of yTop I-Rpc82 complex during the 200 ns simulation (blue) compared with the interaction regions of yTopI (pink) and Rpc82 (green)

Parameter	score
**DOPE**	-79286
**molpdf**	4632.84
**ERRAT**	93.43%
**VerifY3D**	86.35%
**PROCHECK:**	
**Ramachandran favoured**	95.30 %
**Allowed region**	4.70 %
**Generously allowed**	0.0 %
**Disallowed region**	0.0 %

Supplementary Table S1: Quality assessment parameters and corresponding scores used to evaluate the predicted budding yeast Topoisomerase I model.

Domain	Program/Server	ERRAT overall Quality factor	Verify3D Score	Ramachandran param. (PROCHECK)			
				Core %	Addl Allowed %	Generously Allowed %	Dis allowed %
D1	Ab-initio/Robetta	99.24	87.87	91.7	7.6	0	0.8
D3	trRosetta / https://yanglab.qd.sdu.edu.cn/trRosetta/	99.08	59.29	94.2	4.3	1	0.5
D4	Ab-initio/Robetta	97.02	82.57	97.1	2.9	0	0.5
D5	Ab-initio/Robetta	100	87.32	97	3	0	0.5
Full	Advanced Template Based Modelling/ Modeller	93.42	86.35	95.3	4.7	0.0	0.0

Supplementary Table S2: Modeling approaches and quality assessment parameters used for the selection of individual-domain and full-length yTop I models.

yTop I domain	ΔG value (kcal/mol)	Interaction surface area (Å^2)	Preferred docking site at Rpc82
**D1**	-14.2	1646.4	WH2 and WH3
**D2**	-13.6	1440.5	Mainly at WH2 and limited WH3
**D3**	-15.5	1915.5	WH2, WH3 and few residues of WH1
**D4**	-13.2	1392.5	WH2 and WH3
**D5**	-12.7	1313.5	WH2 and WH3

Supplementary Table S3: Results of domain-wise targeted docking of yTop I with Rpc82. Individual yTop I domains predominantly exhibited a preference for docking to the Rpc82 surface formed by the WH2 and WH3 domains. Binding energy calculations were done using PRODIGY server.

D1=141 RESIDUES	PDB ID	ERRAT	Verify3D	PROCHECK			
Selection: 136-146 residues				Most Fav Region	Addl All	Gen All	Disallowed
	1A3N_ChA	96.99	82.98	94.4	5.6	0	0
	1AFC_ChA	89.83	91	76.9	22.2	0.9	0
	1AHQ	87.2	66.17	90.1	9.1	0.8	0
	1ALY	97.1	84.93	81.9	14.2	0	1.6
	1AWI_ChA	87.59	94.93	82.9	16.2	0	0.9
	1B6Z_ChA	86.71	90.58	91.1	8.9	0	0
	1BIN_ChA	96.29	72.03	94.6	5.4	0	0
	1CBQ	96.66	67.15	90.3	8.9	0	0.8
	1CNU	98.4	71.4	92.6	6.6	0	0.8
	1DQE_ChA	98.4	82.4	95.9	4.1	0	0
	1DSY	82	**100**	84	13.4	1.7	0.8
	1E6F_ChA	97.5	72	84.1	15.9	0	0
	1E31_ChA	92.9	89.7	87.5	11.7	0.8	0
	1EAO_ChA	91.3	75.8	86.9	13.1	0	0
	1EJ8	93.1	85	90.9	8.3	0.8	0
	1EW6_ChA	100	84	94.4	5.6	0	0
	1EWX	99	90	92.6	7.4	0	0
	1EXZ_ChA	83.87	61.6	77.7	14.9	5.8	1.7
	1EYH	100	70.8	94.8	5.2	0	0
	1F3H_ChA	93.75	87.5	79.2	19.2	1.7	0
	1FM5	92.7	96.5	89.4	9.6	0	1
	1GAK	100	83.9	94.2	5.8	0	0
	1GP0	96.7	75.9	84.5	15.5	0	0
	1GVJ	98.5	86.5	90.2	9.8	0	0
	1H3Q	91.6	86.4	84.9	13.5	0.8	0.8
	1HCN_Dimr	79.2	3.08	86.4	11.1	1.2	1.2
	1HDK	96.1	74.8	88.7	9.7	0.8	0.8
	1HML	85.5	53.6	85	15	0	0
	1HUP	75.7	59.57	89.6	10.4	0	0
	1I5G	92.42	89.58	91.9	8.1	0	0
	1K9J_ChA	71.9	81.54	88.7	10.4	0.9	0
	1K93_ChD	82.96	93.7	82.8	17.2	0	0
	1KT9	84.25	76.5	89.4	10.6	0	0
	1LCL	98.48	90	89.7	9.5	0	0.8
	5WF1	100	61.95	96.9	3.1	0	0
	5WI4_ChA	91.5	57.6	95.5	4.5	0	0
	5Y8C	86.1	77.6	93.1	6.9	0	0
	9HGH	96.1	85.6	90.6	8.7	0	0.8
	9HHC	99.1	72	97.5	2.5	0	0
	9HQ5_ChA	95.5	62.6	96.6	3.4	0	0
	9ILO_ChA	100	76.6	92.7	7.3	0	0
	9LKI	88	59.2	87	9.6	0.9	2.6
**Average**		**92.16**	**76.78**	**89.24**	**9.95**	**0.41**	**0.35**

Stdev		7.14	16.58	5.24			
	**D1**	**99.24**	78.87	**91.7**	**7.6**	**0**	**0.8**

Supplementary Table S4: Structural quality assessment of predicted D1 domain of yTop I and collected sample proteins, which have high-resolution structures in RCSB-PDB database. [Abbreviations: Most Fav Region- Ramachandran Most favoured region, Addl All – Additionally allowed, Gen All-Generously Allowed]

**D3: 226 residues**	**PDB ID/Protein**	**ERRAT**	**Verify3D**	**PROCHECK**			
**selection: 221-231 residue**				**Most Fav Region**	**Addl All**	**Gen All**	**Disallowed**
	1A8L	99	**53.9**	93.1	6.9	0	0
	1BYQ	96	**73.7**	88.5	8.9	2.6	0
	1DPR_ChA	94.4	**51.4**	79.7	18.2	2.1	0
	1EM2	84	**67**	93.1	6.9	0	0
	1F7C	98.8	**56.5**	90.6	9.4	0	0
	1FBN	92.3	**62.6**	85.6	14.4	0	0
	1FLL_ChA	45.9	**64.3**	91	9	0	0
	2AFR	98.55	**48.1**	90.2	9.8	0	0
	2CHG_ChA	97.2	**71.3**	95.4	4.6	0	0
	2DFH	96.1	**62.4**	89.3	9.6	0.5	0.5
	2IAK	94.2	**53**	84.9	13.8	0.7	0.7
	2VGE	96.93	**60.39**	94.4	5	0.6	0
	3OK8_ChA	91.46	**59.82**	96.2	3.8	0	0
	3T9L	98.1	**69.37**	95	5	0	0
	3ZYQ	95.6	**54.1**	87.8	11.1	1.1	0
	4B9X	83.5	**61.86**	86.5	12.9	0.6	0
	4F9Z_ChA	98.57	**69.41**	89.3	9.5	0.6	0.6
	4FZS_ChA	97	**34.9**	94.6	5.4	0	0
	4HH8	90.16	**61.17**	91.9	7.1	0.5	0.5
	4JK7_ChA	98	**49.32**	95	4.5	0.5	0
	4K51_ChA	95.6	**71.36**	92	7.5	0.5	0
	**Average**	**92.45**	**59.80**	**90.67**	**8.73**	**0.49**	**0.11**
	Stdev	11.5	9.5	4.2			
	**D3**	**99.08**	**59.29**	**94.2**	**4.3**	**1**	**0.5**

Supplementary Table S5: Structural quality assessment of predicted D3 domain of yTop I and collected sample proteins, which have high-resolution structures in RCSB-PDB database. [Abbreviations: Most Fav Region- Ramachandran Most favoured region, Addl All – Additionally allowed, Gen All-Generously Allowed]

D4: 109 AA	PDB ID	ERRAT	Verify3D	PROCHECK			
selection range: 104-114 residues				Most Fav Region	Addl All	Gen All	Disallowed
	1C9H	92.92	97.2	86.6	9.3	3.1	1
	1CEW	88.88	40.74	87.4	12.6	0	0
	1CRC_ChA	83.3	100	88.4	10.5	1.2	0
	1DDV	95.7	86.54	87.4	12.6	0	0
	1EFQ	91.83	81.3	89.7	9.3	1	0
	1GH2	100	78.5	93.3	6.7	0	0
	1GV2	94.68	86.27	83.1	15.5	1.4	0
	1H7C	97.8	54.55	93.5	6.5	0	0
	6YR8_ChA	96.7	79.27	92.9	7.1	0	0
	6YWZ_ChA	95.55	65.69	92.2	6.7	1.1	0
	7A5M	95.14	75.8	95.9	4.1	0	0
	7DIW	100	69.52	82	15.6	1.6	0.8
	7EVS_ChA	95.65	46.53	93.9	6.1	0	0
	7JL5_ChA	77.41	84	93.8	6.2	0	0
	7L3L_ChA	90.9	39.72	88	12	0	0
	7LTW_ChA	81.91	52.43	92.3	7.7	0	0
	7M3Y	97	47.71	86.7	13.3	0	0
	7MQQ	96.1	91.15	90	10	0	0
	7MSJ	92.2	21.1	96.7	3.3	0	0
	7NA1_ChA	100	79.7	87.1	11.8	1.2	0
	7NCV	100	62.26	82.6	15.2	2.2	0
	7NLI_ChA	98.76	74.16	92.1	7.9	0	0
	7NY8	93.7	78.5	92.9	7.1	0	0
	7O0B	93.4	79.8	91.1	8.9	0	0
	7O86_ChA	98.83	66.67	91	9	0	0
	7OKW_ChA	97.61	73.2	91.2	8.8	0	0
	7Q5O	97	97.27	91.7	8.3	0	0
	7RPP_ChA	100	70	94	6	0	0
	7V9H	98.92	87.25	95	5	0	0
	7Y5R	92.63	91.43	93	7	0	0
	7YIR	96.59	82.83	93	7	0	0
	8AH7	97.61	85.29	95.1	4.9	0	0
	8CZ9	95.87	87.96	93.2	6.8	0	0
	8HT3	90.62	94.23	93.5	4.3	1.1	1.1
	8OQ9_ChA	77.65	81.55	90.4	9.6	0	0
	8PHY_ChA	84.26	89.32	87.8	11	1.2	0
	8PWC_ChA	96.51	90.43	92.9	7.1	0	0
	8R6I_ChA	94.3	56.14	95	5	0	0
	8RW6	98.97	65	95.9	4.1	0	0
	8RXA	100	55.88	92.6	7.4	0	0
	8T9U_ChF	100	100	92.7	7.3	0	0
	8X2P_ChA	90.5	100	88.3	11.7	0	0
	9C66	100	83.64	88.9	8.9	1.1	1.1
	9DPM	96.7	57.5	94.7	5.3	0	0
	9EH9	100	100	93.3	6.7	0	0
	9IWA	100	82.65	87.9	11	0	1.1

	9J1N	95.3	100	91.9	8.1	0	0
	9N21	98.94	84.47	93	7	0	0
	9ODS_ChA	83.5	69.44	86.2	11.7	1.1	1.1
	9WH0_ChA	87	87	87.9	11	0	1.1
	9Z0M	80	68.81	89.4	10.6	0	0
	**Average**	**94.09**	**76.09**	**90.96**	**8.56**	**0.34**	**0.14**
	Stdev	6.18	17.97	3.49			
	**D4**	**97.02**	**82.57**	**97.1**	**2.9**	**0**	**0**

Supplementary Table S6: Structural quality assessment of predicted D4 domain of yTop I and collected sample proteins, which have high-resolution structures in RCSB-PDB database. [Abbreviations: Most Fav Region- Ramachandran Most favoured region, Addl All – Additionally allowed, Gen All- Generously Allowed]

**PDB ID**	**ERRAT**	**Verify3D**	**Ramach Most Fav**
**1B3A_ChA**	**92.3**	**62.69**	**86.2**
**1CC7**	**100**	**83.33**	**95.3**
**1CKT**	**98.41**	**80.28**	**96.2**
**1CSK_ChA**	**94**	**98.28**	**94.7**
**1TG0**	**100**	**74.24**	**90**
**1YU5**	**100**	**77.61**	**89.7**
**1ZU3**	**83.92**	**68.75**	**90.4**
**2OCH**	**100**	**100**	**91.2**
**2Y9U**	**100**	**92.5**	**86.8**
**3B32**	**90**	**98.65**	**96.6**
**3CJK_ChA**	**100**	**86.76**	**96.6**
**3IWL**	**100**	**100**	**87.7**
**3SD4_ChA**	**100**	**100**	**83.3**
**4A3N**	**96.49**	**71.88**	**94.8**
**4BMJ_ChA**	**80.39**	**74.19**	**93.1**
**4CC3_ChA**	**100**	**60.66**	**88.1**
**4OU0**	**98.24**	**100**	**96.2**
**4PLI_ChA**	**97.87**	**98.25**	**95.2**
**4RBO_ChA**	**100**	**94.44**	**91.5**
**5E8B_ChA**	**100**	**74.63**	**93.3**
**5U6Z**	**69.49**	**63.89**	**91.9**
**5UM3**	**100**	**90.63**	**91.9**
**5VNY**	**92.85**	**100**	**96.2**
**D5**	**100**	**87.32**	**97**

Supplementary Table S7: Structural quality assessment of predicted D5 domain of yTop I and collected sample proteins, which have high-resolution structures in RCSB-PDB database. [Abbreviations: Ramach Most Fav - Ramachandran Most favoured region, Addl All – Additionally allowed, Gen All-Generously Allowed]

Full-length 769 residues		PDB ID/Protein	ERRAT	Verify3D	PROCHECK			
selection:740-780 residues					Most Fav Region	Addl All	Gen All	Disallowed
	**Modeller**	**yTop I**	**93.43**	**86.35**	**95.3**	**4.7**	**0**	**0**
		1H2V_ChC	93.97	81	85.2	12.6	1.8	0.3
		1U1H	93.4	82.5	93.2	6.6	0.2	0
		1W8J_ChA	89.73	85	87.9	10.7	1	0.4
		3EH2_ChA	86.11	77.7	86.9	11.9	1.1	0.2
		3P5P	98.23	92.21	87.9	12.1	0	0
		3TV5_ChA	93.47	75	86.4	12.3	0.8	0.5
		3ZVR	89.93	73.24	90.9	8.7	0.2	0.2
		5JPT_ChA	95.63	77.58	85	14.7	0.3	0
		6AFS_ChA	99.58	50.14	93.7	5.9	0.3	0.1
		6NS3	88.31	83.57	96.9	3.1	0	0
		6NS5_ChA	96.28	84.13	92.8	6.9	0.2	0.2
		7DHW	91.19	84.71	86.7	13	0.1	0.1
		7JTN_ChA	86.89	93.87	93.2	6.7	0.2	0
		7R2X_ChA	93.58	91.34	83.9	15.3	0.8	0
		8HR0_ChA	85.53	71	83.9	15.3	0.8	0
	**Type IB**	1OIS_yTop I	89.67	95	92.7	6.8	0.6	0
		2B9S_L_donov	94.4	72.59	92.1	7.9	0	0
		1EJ9_hTop1	90.7	86.54	90.8	8.9	0	0.2
		1K4T_hTop1	94.23	84.75	93	5.9	1.1	0
		**Average**	**92.15**	**81.15**	**89.64**	**9.75**	**0.50**	**0.12**
		stdev	3.96	10.35	3.90			

Supplementary Table S8: Structural quality assessment of the predicted yTop I model and reference proteins with high-resolution structures available in the RCSB Protein Data Bank (PDB), including human and *Leishmania* Topoisomerase I structures. [Abbreviations: Most Fav - Ramachandran Most favoured region, Addl All – Additionally allowed, Gen All- Generously Allowed]

Server/Prog.	PDB ID/Name	ERRAT	Verify3D	PROCHECK				QMEAN
				Most Fav Region	Addl All	Gen All	Disallowed	
**Modeller**	**yTop I**	**93.43**	**86.35**	**95.3**	**4.7**	**0**	**0**	**0.16**
**Galaxy**	yTp1_TBM_1	92.51	70	93.8	5.2	0.4	0.6	**-0.65**
**Galaxy**	yTp1_TBM_2	90.16	65	93.5	5.7	0.3	0.6	-0.68
**Galaxy**	yTp1_TBM_3	89.44	66.97	93.8	5.1	0.4	0.7	-0.51
**Galaxy**	yTp1_TBM_4	91	68.27	94	5.1	0.3	0.6	-0.64
**Galaxy**	yTp1_TBM_5	89.87	64.24	93.5	5.7	0	0.9	-0.62
**Protenix**	Model_1	90.32	77.24	86.8	12.1	1.1	0	**-0.32**
**Protenix**	Model_2	93.11	80.49	84.5	13.6	1.8	0	-0.26
**Protenix**	Model_3	94.3	77.5	84.5	13.9	1.6	0	-1.11
**Protenix**	Model_4	94.76	74.64	89.1	10.6	0.3	0	-0.58
**Protenix**	Model_5	96.69	73.34	86.2	11.1	2.7	0	0.8
**AlphaFold**	AF-P04786-F1	**94.43**	**75.29**	**86.2**	**10.9**	**1**	**1.8**	-1.08

Supplementary Table S9: Structural quality assessment of the predicted yTop I model and models generated using AlphaFold, GalaxyWEB, and the Protenix server. The colour gradient indicates the relative quality of the corresponding structural validation metrics, with green representing more favourable values compared with the other values within each assessment category. [Abbreviations: Most Fav Region- Ramachandran Most favoured region, Addl All – Additionally allowed, Gen All- Generously Allowed]

Model	No. of Interaction	% Interaction calculated based on domain specific No. of interaction (including hydrophobic int.)						ΔG
		NTD	WH1	WH2	WH3	WH4	CC	
Cluspro_1	25	40.0	24.0	0.0	0.0	24.0	12.0	-8.9
Cluspro_2	27	25.9	29.6	0.0	0.0	22.2	22.2	-13.9
Cluspro_3	58	0.0	0.0	96.6	3.4	0.0	0.0	1.9
Cluspro_4	47	0.0	36.2	6.4	8.5	17.0	31.9	-2.2
Cluspro_5	44	0.0	29.5	0.0	0.0	68.2	2.3	-7.9
Cluspro_6	40	7.5	12.5	7.5	12.5	60.0	0.0	-15.9
Cluspro_7	45	0.0	4.4	0.0	0.0	0.0	95.6	-3.3
Cluspro_8	32	21.9	40.6	0.0	0.0	28.1	9.4	-12.6
Cluspro_9	39	0.0	0.0	64.1	35.9	0.0	0.0	-11.5
Cluspro_10	49	22.4	20.4	0.0	0.0	38.8	18.4	-21.6
HDOCK_1	23	0.0	34.8	17.4	8.7	8.7	30.4	-6.1
HDOCK_2	9	0.0	0.0	0.0	33.3	66.7	0.0	-9.2
HDOCK_3	19	0.0	10.5	0.0	0.0	36.8	52.6	-9.1
HDOCK_4	13	15.4	7.7	0.0	0.0	76.9	0.0	-8.3
HDOCK_5	21	0.0	0.0	0.0	47.6	47.6	4.8	-9.3
HDOCK_6	22	9.1	0.0	0.0	0.0	45.5	45.5	2.5
HDOCK_7	17	0.0	0.0	0.0	52.9	47.1	0.0	-7.4
HDOCK_8	20	0.0	0.0	85.0	15.0	0.0	0.0	-1.1
HDOCK_9	17	0.0	11.8	70.6	17.6	0.0	0.0	-10.2
HDOCK_10	18	0.0	0.0	0.0	44.4	55.6	0.0	-14
HADDOCK_1	19	0.0	10.5	21.1	10.5	21.1	36.8	-0.5
HADDOCK_2	18	0.0	33.3	61.1	5.6	0.0	0.0	-4.4
HADDOCK_3	17	0.0	0.0	0.0	100.0	0.0	0.0	-8.3
HADDOCK_4	14	0.0	0.0	100.0	0.0	0.0	0.0	-1.9
HADDOCK_5	17	0.0	76.5	23.5	0.0	0.0	0.0	-3.8
HADDOCK_6	20	0.0	55.0	0.0	0.0	0.0	45.0	-8.8
HADDOCK_7	14	0.0	7.1	50.0	42.9	0.0	0.0	1.4
HADDOCK_8	23	0.0	0.0	65.2	34.8	0.0	0.0	-5.8
HADDOCK_9	25	0.0	0.0	0.0	28.0	40.0	32.0	-4.4
HADDOCK_10	27	0.0	0.0	66.7	33.3	0.0	0.0	-0.7
Rpc82_34 (PDB)	15	0.0	0.0	0.0	13.3	53.3	33.3	-24.4
Rpc82_160 (PDB)	39	0.0	33.3	0.0	0.0	56.4	10.3	-13.9
Model Frequency		7	17	14	18	17	13	

Supplementary Table S10: PDBePISA analysis of the molecular docking interface between full-length yTop I and Rpc82, showing the predicted interaction energy (ΔG, kcal/mol). Ten top scoring models from each server were selected for analysis. Total number of interactions are mentioned in the second column. Interaction of yTop I at different domains of Rpc82 surface was estimated and converted to percentage values to determine the contribution of each domain of Rpc82 towards the total binding interactions observed with yTop I. In few models, yTop I was observed to bind at WH1-WH4-CC surface, which are shaded in green colour whereas models wherein WH2-WH3 surface of Rpc82 formed the binding surface are shaded in violet color. Column with ΔG values have also been shaded with different colours, where blue indicated better (lowest values) binding free energies and red indicated higher values. Interaction between Rpc82-Rpc34 and Rpc82-Rpc160 were also measured (collected from PDB ID: 6EU0) and mentioned at final two rows.

Sl. No.	PDB ID	Binding energy<br>kcal/mol	kcal/mol/Å^2<br>Energy/Surface Area
1	1A22	-15.7	-0.012
2	1DJ7	-10.7	-0.014
3	1E44	-10.9	-0.009
4	1H2V	-12.3	-0.010
5	1H32	-11.4	-0.009
6	1KA9	-11.3	-0.008
7	1OF5	-12.5	-0.009
8	1OO0	-10.5	-0.009
9	1UGH	-15.3	-0.014
10	1WPX	-13.5	-0.008
11	2APO	-9.6	-0.008
12	2B42	-13	-0.010
13	2D74	-11.3	-0.008
14	2OMZ	-17.3	-0.013
15	2P1M	-11.6	-0.007
16	2RAW	-7.4	-0.005
17	2VSM	-12	-0.009
18	2XFG	-14.2	-0.013
19	2Z5B	-12.2	-0.011
20	2Z64	-13.6	-0.012
21	2ZSI	-11.4	-0.009
22	3AWU	-12.6	-0.011
23	3CPT	-12	-0.010
24	3CX8	-12.9	-0.009
25	3H7H	-12.4	-0.008
26	3IEY	-11.6	-0.008
27	3KXC	-8	-0.008
28	3MXN	-11.9	-0.009
29	3N4I	-12	-0.009
30	3SHG	-11.7	-0.008
31	3ZYI	-11.1	-0.011
32	4BL7	-10.6	-0.008
33	4CBU	-10.7	-0.010
34.	yTopI-Rpc82 complex	-12.7	-0.008

Supplementary Table S11: Established Heterodimeric proteins of similar size and interaction surface area compared with the selected Top1-Rpc82 complex in terms of binding energy. Data were collected from RCSB-PDB database. Binding energies were calculated using PRODIGY server to prepare the standard plot.

0ns		20 NS		40 NS		60 ns		80 ns		100 ns		120 ns		140 ns		160 ns		180 ns		200 ns	
Rpc82	Top1	Rpc82	Top1	Rpc82	Top1	Rpc82	Top1	Rpc82	Top1	Rpc82	Top1	Rpc82	Top1	Rpc82	Top1	Rpc82	Top1	Rpc82	Top1	Rpc82	Top1
R145	D50	R145	P46	R145	L40	R145	D42	R145	L40	R145	L40	R145	D42	D138	D5	D138	D42	D138	D42	R145	D42
V146	E51	V146	D50	V146	D42	V146	E43	V146	D42	V146	D42	V146	E45	E139	E12	E139	E43	E139	E43	V146	P46
D148	K58	D148	K123	D148	E43	N147	P46	D148	E43	D148	E43	E149	P46	R145	D16	R145	E45	V146	E45	D148	D48
E149	K62	E149	K125	E149	P46	D148	Y47	E149	P46	E149	E45	E150	D50	V146	D42	V146	P46	D148	P46	E149	E51
E150	A124	E151	E126	E151	Y47	E149	D50	E151	Y47	E151	P46	D181	E51	D148	E43	E149	E43	E149	Y47	E150	K123
H152	K125	H152	E127	D181	D50	E151	E51	S182	D50	Y185	Y47	E196	E126	E149	E45	E150	D48	E150	D48	E151	E126
Y185	E126	D181	E128	S182	K62	D181	K62	Y185	E51	T186	D50	K221	E127	E150	P46	E151	D50	E151	D50	D181	E127
V192	E127	S182	E130	Y185	K63	S182	K122	T186	S54	S189	S54	N225	E130	E151	D48	E158	E130	E158	E120	E196	E128
E196	E128	Y185	S384	T186	I64	Y185	K123	S189	K123	V192	K62	R228	A298	E158	E51	D181	E135	E172	E126	K221	E129
K251	Y385	T186	Y385	S189	E116	T186	K125	V192	E126	Q193	K125	K251	R296	D173	K123	E196	E139	D173	E127	N225	E139
K255	A388	V192	A388	V192	K123	S189	E126	Q193	E127	E196	E126	K255	Y385	D181	E135	K224	D209	D181	D209	P227	D209
L259	R391	K221	R391	Q193	K125	V192	E127	E196	E130	Q202	E127	S260	K400	E196	E139	N225	A298	K224	A298	R228	A298
S260	D392	K224	D392	E196	E127	Q193	E128	Q202	S384	K221	S384	Q261	V401	P227	A298	P227	D339	N225	P300	K255	D339
Q261	R395	K255	R395	K221	E128	E196	E129	K221	Y385	H222	Y385	T264	M402	R228	D339	R228	Y385	P227	K400	Q261	E350
L263	K400	L259	K400	K224	E130	Q202	E130	K224	A388	K224	D392	V265	E424	K251	K400	P267	S426	R228	V401	D266	K400
T264	V401	S260	V401	P227	S384	K221	S384	I226	D392	N225	R395	P267	S426	D266	V401	T269	E427	D266	L403	P267	V401
V265	M402	Q261	L403	R228	Y385	H222	Y385	P227	R395	P227	K400	T269	E427	P267	L403	S270	D431	P267	S426	T269	L403
D266	L403	I262	E404	N244	A388	K224	A388	R228	K400	R228	V401	S270	D431	S270	S426	L271	C436	K268	D431	L271	E424
T269	G423	L263	K425	D248	R391	R228	R391	N244	V401	N244	L403	L271	E441	L271	D431	D285	E441	L271	C436	K275	D431
S270	E424	T264	S426	K251	D392	M243	D392	K251	M402	K255	E424	R272	L456	R272	E441	E320	Q486	R272	S437	N362	E441
L271	K425	D266	E427	I252	R395	N244	R395	K255	L403	L259	S426	K275	P487	E320	P487	N362	P487	E320	E441	I364	Q486
R272	S426	P267	P484	K255	K400	D248	K400	L259	E404	S260	E427	N362	G488	N362	G488	I364	G488	I364	Q486	D365	G488
I364	E427	T269	R494	E258	V401	K251	V401	Q261	E424	Q261	G488	I364	H489	I364	Q490	R368	H489	R368	P487	H369	H489
A367	P484	S270	N506	L259	L403	K255	L403	L263	K425	T264	H489	D365	D493	R368	L495	A372	Q490	A372	G488	A372	Q490
R368	K485	L271	Y507	Q261	E404	L259	E404	T264	S426	D266	Q490	R368	L495	H369	D496	E373	D493	E373	H489	E373	R494
H369	Q486	R272	P509	T264	E424	Q261	E424	V265	E427	T269	D493	H369	D496	A372	P497	D375	L495	L374	Q490	D375	D496
L370	G488	K275		D266	K425	L263	E427	D266	Q486	S270	R494	A372	P497	E373	I499	R377	D496	R377	D496	R377	P497
P371	H489	A372		P267	S426	T264	R439	P267	P487	L271	I499	D375	G505	R377	N506	H448	P497	H448	P497	A450	I499
A372	Q490	D375		K268	E427	D266	E441	T269	D493	R272	K502	H448	Y507	H448	Y507	A450	N506	A450	I499	H448	Y507
E373	D493	R377		T269	P484	P267	Q486	S270	R494	K275	N506	A450	P509	A450	P509	E649	Y507	E649	N506		P509
L374	R494	H448		S270	Q486	T269	P487	L271	D496	T277	Y507						P509		Y507		
										D375									P509		
										R377											
										H448											
										S451											
										N454											

Special patch developed after initiation of simulation

Interacting residues of Rpc82 showing sustained involvement over a prolonged period of simulation

Interacting residues of TopI showing sustained involvement over a prolonged period of simulation

Supplementary Table S12: Identification of key amino acid residues forming the binding interface between the two proteins (yeast Topoisomerase I and Rpc82 subunit of RNA polymerase III) during MD simulation. These residues seem to be involved in forming the binding interface between the two proteins throughout the simulation period. The amino acid residues of yTop I, which seem to maintain contact with the partner protein Rpc82 almost throughout the simulation period have been highlighted in yellow. On the other hand, amino acid residues of Rpc82, which seem to maintain contact with the partner protein yTop I for a prolonged period of the simulation have been highlighted in blue. Interacting residues that emerge during the course of simulation have been depicted in pink colour.

All data supporting the findings of this study are available in a Zenodo repository and accessible through Zenodo DOI (https://zenodo.org/records/21863991**)**
